# Targeting Intratumoral Copper Inhibits Tumor Progression via p62‐Mediated EZH2 Degradation and Potentiates Anti‐PD‐1 Immunotherapy in Oral Squamous Cell Carcinoma

**DOI:** 10.1002/advs.202417795

**Published:** 2025-07-28

**Authors:** Xiaohu Lin, Wanling Chen, Bo Li, Zhang Zhao, Zhonglin Yu, Xu‐Yun Zhao, Xuan Zhou, Zhien Feng, Chengzhong Lin, Wei Cao

**Affiliations:** ^1^ Department of Oral Maxillofacial‐Head and Neck Oncology Shanghai Ninth People's Hospital Shanghai Jiao Tong University School of Medicine College of Stomatology Shanghai Jiao Tong University No. 639, Zhizaoju Rd Shanghai 200011 China; ^2^ National Center for Stomatology National Clinical Research Center for Oral Diseases Shanghai 200011 China; ^3^ Shanghai Key Laboratory of Stomatology Shanghai 200011 China; ^4^ Department of Oral Pathology Shanghai Ninth People's Hospital Shanghai Jiao Tong University School of Medicine, College of Stomatology Shanghai Jiao Tong University No. 639, Zhizaoju Rd Shanghai 200011 China; ^5^ Department of Oral and Maxillofacial‐Head and Neck Oncology Beijing Stomatological Hospital, Capital Medical University Beijing 100070 China; ^6^ Department of Biochemistry and Molecular Cell Biology Shanghai Key Laboratory for Tumor Microenvironment and Inflammation Key Laboratory of Cell Differentiation and Apoptosis of the Chinese Ministry of Education Shanghai Jiao Tong University School of Medicine Shanghai 200025 China; ^7^ Department of Maxillofacial and Otorhinolaryngological Oncology Tianjin Medical University Cancer Institute & Hospital Tianjin 300060 China; ^8^ State Key Laboratory of Druggability Evaluation and Systematic Translational Medicine Tianjin 300060 China; ^9^ Department of Oral and Maxillofacial Surgery Zhongshan Hospital Fudan University No.180, Xietu Rd Shanghai 200011 China

**Keywords:** EZH2, Immunotherapy, Intratumoral copper, OSCC, p62

## Abstract

High copper levels are required for tumor initiation and progression, termed cuproplasia. However, its role and underlying mechanisms in oral squamous cell carcinoma (OSCC) remain poorly understood. It is find that copper and its transporter SLC31A1 accumulate extensively in OSCC. Blocking copper influx through SLC31A1 siRNA or copper chelators significantly represses OSCC both in vitro and in vivo. Single‐cell RNA sequencing and multiplex immunohistochemical staining revealed that copper chelators specifically decrease the stem‐like tumor epithelial cell subpopulation. Further investigation shows that intratumoral copper depletion reduces histone methyltransferase EZH2 expression at the protein level, but not at the mRNA level, as determined through a screen analysis of histone‐modification enzymes. Mechanistically, it is discovered that silencing SLC31A1 and treating with copper chelators increase p62‐mediated EZH2 ubiquitination at the Ub‐K63 site by suppressing copper binding to SMURF2, an E3 ligase of EZH2, leading to its autophagic degradation. Additionally, combining copper chelators with anti‐PD‐1 treatment effectively suppresses tumor growth, and high levels of SLC31A1 are notably associated with non‐response to anti‐PD‐1 treatment. In conclusion, the crucial role of copper in modulating EZH2 protein stability is demonstrated, and a new approach using copper chelators and anti‐PD‐1 therapy for OSCC patients is provided.

## Graphical abstract

The authors find that by targeting intratumoral copper, they can enhance p62‐mediated ubiquitination of EZH2 at the Ub‐K63 site by suppressing copper binding to SMURF2, an E3 ligase of EZH2, leading to its autophagic degradation. This mechanism suppressed OSCC progression and potentiated anti‐PD‐1 immunotherapy, highlighting a potential new therapeutic strategy.

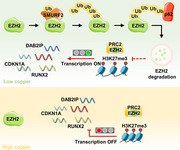

## 1 Introduction

Oral squamous cell carcinoma (OSCC) represents the most prevalent subtype of head and neck squamous cell carcinoma (HNSCC), exhibiting high incidence and mortality rates worldwide.^[^ ^1^ ^]^ The 5‐year overall survival of advanced OSCC patients remains poor due to the complexity and uncertainty of OSCC pathogenesis. Although genomic and epigenetic alterations partially contribute to the worse survival of OSCC patients,^[^ ^2^, ^3^ ^]^ the pathogenesis of OSCC is still far from understood. Recently, more attention has been paid to the role of copper in promoting carcinogenesis.^[^ ^4^ ^]^

Copper homeostasis plays a critical role in various physiopathological processes.^[^ ^5^ ^]^ The impact of copper on cellular function is dose‐dependent. High copper levels can promote cell proliferation, while excessive copper exposure leads to cuproptosis.^[^ ^6^ ^]^ For instance, bioinformatic analysis of the TCGA dataset revealed widespread upregulation of SLC31A1, which encodes a well‐known copper transport protein facilitating copper uptake across multiple cancer types,^[^ ^7^, ^8^ ^]^ indicating cancer cell proliferation and growth dependent on intracellular copper. Studies have also demonstrated that chronic copper exposure or daily oral copper supplementation accelerates tumor growth in animal models of pancreatic islet cell carcinoma and breast cancer.^[^ ^9^, ^10^ ^]^ Mechanistically, copper exerts its pro‐tumorigenic effects through multiple pathways. It serves as a cofactor for mitochondrial proteins MT‐CO1 and MT‐CO2, thereby promoting cancer cell proliferation.^[^ ^11^ ^]^ In addition, copper acts as a cofactor for MEK1 and MEK2, enhancing their ability to phosphorylate ERK1 and ERK2, which in turn stimulates tumor growth through the MAPK signaling pathway.^[^ ^12^ ^]^

Notably, copper promotes the ubiquitination proteasome pathway for the degradation of UPS‐mediate tumor suppressor proteins such as p53, CRIP2, and IκB.^[^ ^13^, ^14^, ^15^ ^]^ Copper also facilitates the assembly of autophagy machinery, which can support cancer cell proliferation, as demonstrated in a model of KRAS‐mutant lung adenocarcinoma.^[^ ^16^ ^]^ On the other hand, excessive copper accumulation induces cell cuproptosis.^[^ ^17^ ^]^ The complex interplay between copper's tumor‐promoting and tumor‐repressive effects underscores the delicate balance of copper homeostasis in cancer biology, highlighting the potential for copper‐targeted therapies in cancer treatment.

Enhancer of zeste homolog 2 (EZH2) serves as the catalytic subunit of the polycomb repressive complex 2 (PRC2), where it primarily methylates lysine‐27 of histone H3. This modification is well‐known for its role as a suppressor of gene transcription, contributing to cell differentiation, the maintenance of adult stem cell populations, and tumorigenesis.^[^ ^18^ ^]^ Interestingly, EZH2 also functions as a transcriptional co‐activator, promoting carcinogenesis through its non‐canonical roles.^[^ ^19^ ^]^ This dual functionality underscores the importance of targeting EZH2 in solid tumors as a potential therapeutic strategy. Despite the approval of Tazemetostat (EPZ6438) by the FDA in 2020 as an EZH2 inhibitor, its clinical efficacy in most solid tumors has not met expectations. This shortcoming suggests that solely targeting EZH2's enzymatic activity may be insufficient.^[^ ^20^ ^]^ In response, recent interest has shifted toward targeting EZH2 degradation, which may yield more robust therapeutic effects. However, existing studies on EZH2 degradation in cancer cells have predominantly centered on the ubiquitin‐proteasome system.^[^ ^20^ ^]^ Further investigation is warranted to determine whether alternative degradation mechanisms for EZH2 are involved, particularly in the context of OSCC. Understanding these mechanisms could provide valuable insights for enhancing the therapeutic targeting of EZH2 in cancer treatment.

The introduction of immune checkpoint inhibitors (ICIs) has significantly transformed HNSCC therapy, particularly those targeting programmed cell death 1 (PD‐1) and its ligand programmed death‐ligand 1 (PD‐L1). Clinical evidence demonstrates that these ICIs have contributed to a partial improvement in patient survival rates.^[^ ^21^, ^22^ ^]^ However, the efficacy of anti‐PD‐1 monotherapy remains limited in advanced and metastatic HNSCC patients. The objective response rate (ORR) for this treatment modality is disappointingly low, ranging from only 18% to 34%,^[^ ^23^, ^24^ ^]^ suggesting a majority of OSCC patients do not benefit from the treatment. This suboptimal response rate underscores the pressing need for innovative combined therapeutic strategies.

In this study, we uncovered a novel mechanism linking intracellular copper deficiency to the regulation of EZH2 stability in OSCC. Our findings demonstrated that targeting cuproplasia significantly suppressed the malignant phenotypes of OSCC cells. Importantly, we have found that p62 directly binds EZH2 and leads to EZH2 ubiquitination‐mediated selective autophagic degradation in the nucleus. Moreover, we found that reducing intracellular copper levels disrupts the binding between copper and SMURF2, an E3 ubiquitin ligase of EZH2. This disruption leads to the stabilization of SMURF2, which in turn promotes p62‐mediated K63‐linked ubiquitination and subsequent degradation of EZH2. Finally, our results demonstrated that copper chelator‐TEPA restores sensitivity to anti‐PD‐1 therapy by enhancing tumor‐infiltrating T lymphocytes.

## 2 Results

### 2.1 Serum Copper Level and SLC31A1 Indicated Cancer Progression and Poor Survival in OSCC and Human Cancers

**Figure 1:**
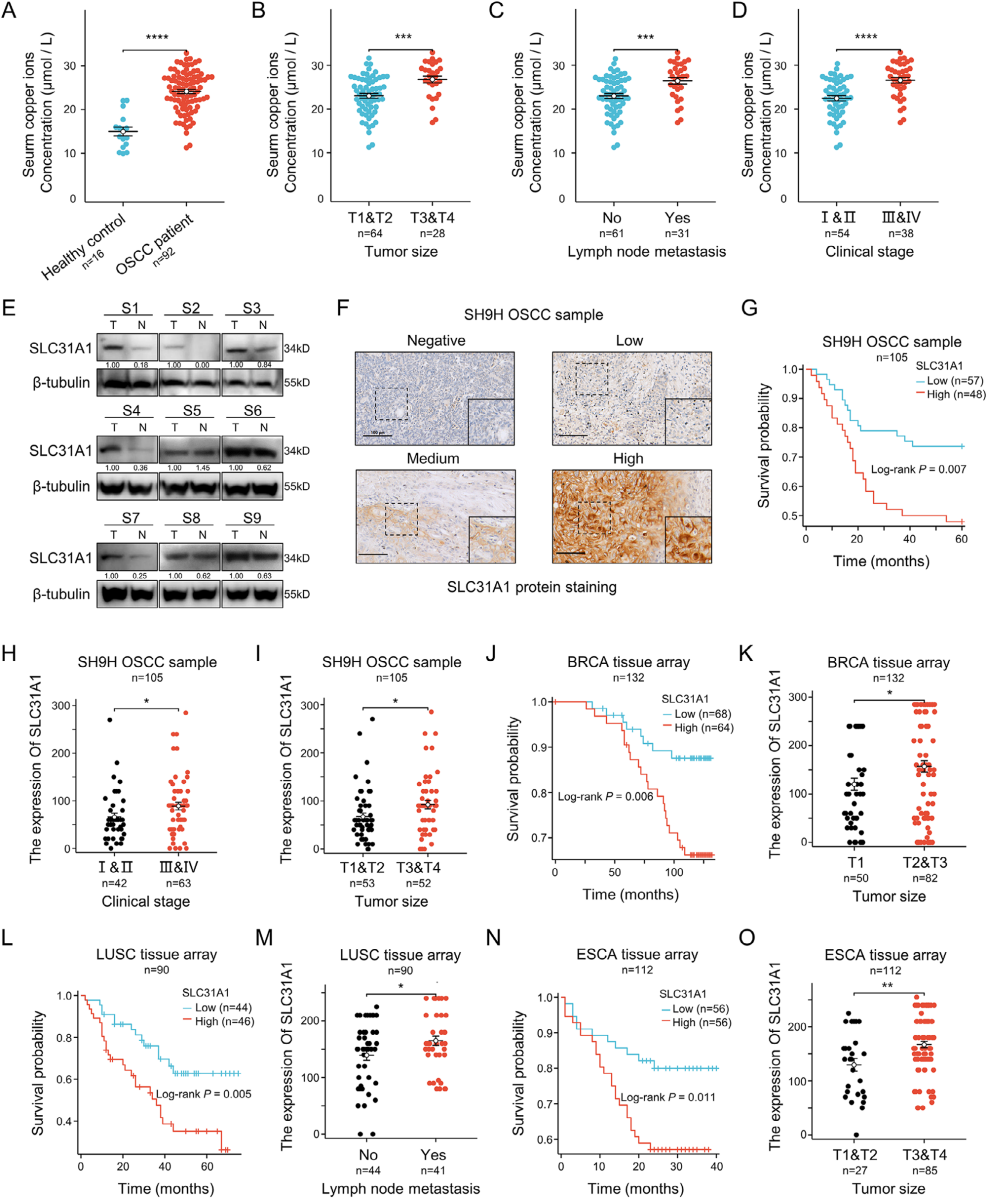
Serum copper level and SLC31A1 indicated cancer progression and poor survival in OSCC and human cancers. A) Serum copper ion levels in OSCC patients (n = 92) were compared with those in a healthy control group (n = 16). B–D) The relationship between serum copper ion levels and tumor size (B), lymph node metastasis (C) and tumor clinical staging (D) in OSCC patients were analyzed. E) Expression levels of SLC31A1 protein in 9 pairs of OSCC tumor tissues and adjacent normal tissues. F–I) In an independent clinical sample of OSCC from the Shanghai Ninth People's Hospital (n = 105) (F), the correlation between SLC31A1 expression and tumor clinical staging (H), tumor size (I), and patient prognosis (G) were analyzed, Scale bars, 100 µm. J,K) In BRCA tissue microarrays (n = 132), the correlation between SLC31A1 expression and tumor size (K), as well as patient prognosis (J), were analyzed. L,M) In LUSC tissue microarrays (n = 85), the association between SLC31A1 expression and lymph node metastasis (M), as well as patient prognosis (L), were analyzed. N,O) In ESCA tissue microarrays (n = 112), the correlation between SLC31A1 expression and tumor size (O), as well as patient prognosis (N), were analyzed. Data in A‐D were calculated by two‐tailed unpaired Student's t test; Data in H, I, K, M, and O were calculated by the Wilcoxon rank‐sum test; Data in G, J, L, and N were analyzed by Kaplan–Meier plots, p values were determined by a two‐tailed log‐rank test.

Copper is an essential nutrient that is increasingly involved in cell proliferation and death pathways. Therefore, our study investigated the role of copper in tumor progression, mainly focusing on OSCC. A comprehensive approach, from sample collection to data analysis, was conducted to elucidate the relationship between copper homeostasis and OSCC (Figure S1A, Supporting Information). We found that serum copper ion concentration in OSCC patients was significantly higher than in healthy controls, which is associated with OSCC onset and progression (**Figure** **1**A). Elevated levels of serum copper ion were found to be notably related to greater tumor size, lymph node metastasis (LNM), and advanced clinical stage (Figure 1B–D). Subsequently, we detected the expression of SLC31A1 in 9 randomly selected paired OSCC and adjacent normal tissues, and upregulation of SLC31A1 was found in 88.9% (8/9) paired OSCC samples by western blotting assay (Figure 1E). To test the association between SLC31A1 and clinicopathological parameters as well as the overall survival of OSCC patients, the expression levels of SLC31A1 were further confirmed in the two independent OSCC cohorts, respectively, from Shanghai Ninth People's Hospital (SH9H) (n = 105) (Figure 1F–I) and Stomatology Hospital of Capital Medical University (CMU) (n = 247) (Figure S1B–E, Supporting Information). Overexpression of SLC31A1 was remarkably associated with advanced clinical stage, greater tumor size, and worse overall survival of patients from the two independent OSCC cohorts by immunohistochemistry (IHC). However, pan‐cancer analysis showed significant expression differences and prognostic values of SLC31A1 across multiple tumor types based on the TCGA database (Figure S2A–C, Supporting Information). Consistent with the above results, high SLC31A1 expression, detected by IHC, was also found to be markedly associated with greater tumor size, LNM, and adverse overall survival of patients in breast cancer (BRCA), esophageal cancer (ESCA), and lung squamous cell carcinoma (LUSC) (Figure 1J–O; Figure S2D–F, Supporting Information), suggesting the pivotal role for copper in tumor development and indicating SLC31A1 serves as a robust biomarker across various cancer types, underscoring the potential significance of copper homeostasis in tumor biology and its promise as a potential therapeutic target.

### 2.2 Decreasing Intratumoral Copper Abrogated OSCC Cell Growth In Vitro and In Vivo

**Figure 2:**
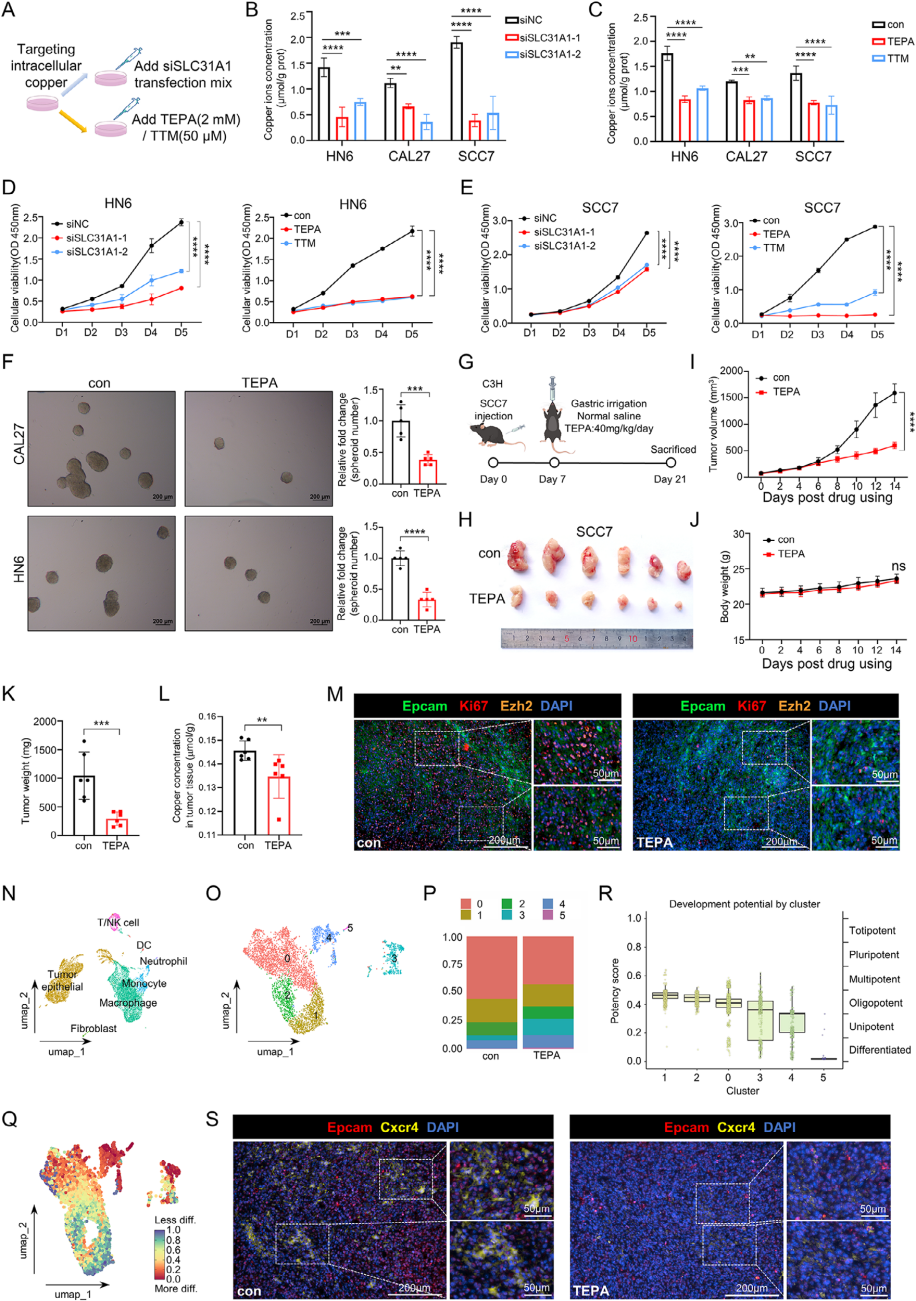
Decreasing intratumoral copper abrogated OSCC cell growth. A) Targeting intratumoral copper by silencing SLC31A1 or using copper ion chelators. B,C) Measurement of cellular copper ion concentrations in OSCC cells after silencing SLC31A1 (B) and using copper ion chelators (C). D,E) The effect of silencing SLC31A1 (D) and copper ion chelators (E) on cell proliferation ability in OSCC cell lines (HN6 and SCC7). F) The effect of copper ion chelators on sphere formation (sphere number) in OSCC cell lines (HN6 and CAL27), Scale bars, 200 µm. G) Schematic diagram of subcutaneous tumor (SCC7) and oral administration experiment in C3H mice. H) Macroscopic view of subcutaneous xenografts in C3H mice. I) Volume changes of subcutaneous xenografts in C3H mice. J) Changes in total body weight of C3H mice. K) Weight of subcutaneous xenografts in C3H mice. L) Copper concentration in tumor tissue. M) Multiplexed immunofluorescence of Epcam, Ki67 and Ezh2 in subcutaneous xenograft tissues from C3H mice, Scale bars, 200 µm (left), 50 µm (right). N) scRNA‐seq analysis of subcutaneous xenograft tumors in C3H mice. O) Dimensionality reduction clustering analysis of tumor epithelial cells in subcutaneous xenograft tumors in C3H mice. P) Proportions of various tumor epithelial cell clusters. Q) CytoTRACE analysis of tumor epithelial cell. R) Development potential of tumor epithelial cell clusters. S) Multiplexed immunofluorescence of Cxcr4 and Epcam in subcutaneous xenograft tissues from C3H mice, Scale bars, 200 µm (left), 50 µm (right). Data were calculated by two‐tailed unpaired Student's t test.

To investigate the effects of targeting cuproplasia in OSCC cells, we employed two strategies: SLC31A1 siRNAs and copper chelators tetraethylenepentamine pentahydrochloride (TEPA)^[^ ^22^ ^]^ or tetrathiomolybdate (TTM)^[^ ^23^ ^]^ (**Figure** **2**A). First, the knockdown effects of SLC31A1‐1 and SLC31A1‐2 siRNAs were evaluated, the result showed that silencing of SLC31A1 significantly reduced SLC31A1 mRNA and protein levels (Figure S3A,B, Supporting Information), leading to a significant decrease in intracellular copper ion concentration in OSCC cell lines (HN6, CAL27 and SCC7) (Figure 2B). Similarly, treating OSCC cell lines with copper chelators TEPA or TTM reduced intracellular copper ion concentration in OSCC cell lines (Figure 2C) without affecting other metal ion concentrations (Figure S3C–F, Supporting Information), indicating the specific targeting of copper ions with copper chelators TEPA or TTM.

Next, we found that reducing intracellular copper remarkably decreases cell proliferation (Figure 2D,E; Figure S3G, Supporting Information), sphere formation (Figure 2F; Figure S3J, Supporting Information) and colony formation ability (Figure S4A,B, Supporting Information), as well as the phosphorylation level of proliferation‐related ERK protein (Figure S4C,D, Supporting Information). The higher sensitivity to copper starvation was observed in OSCC cell lines compared to the human oral normal keratinocyte (HOK) cell line (Figure S3H,I, Supporting Information), suggesting cuproplasia is more specific in tumor providing the potential of target therapy using the copper chelators in OSCC. Additionally, we generated the SLC31A1‐KO HN6 cell line and evaluated its proliferative capacity and colony‐forming efficiency, observing results consistent with those obtained using siSLC31A1 (Figure S4E–G, Supporting Information).

Given the decrease of cell proliferation by reducing intracellular copper on OSCC in vitro, we further investigated its effects on OSCC xenograft tumor growth in vivo. Our results demonstrated that silencing of SLC31A1 significantly reduced the volume and weight of OSCC subcutaneous xenografts, as well as the downregulation of proliferation‐related markers (Ki67 and EZH2) in immunocompromised nude mice (Figure S5A–F, Supporting Information) and immunocompetent C3H mice (Figure S5G–L, Supporting Information). Similarly, treatment with TEPA achieved consistent effects with SLC31A1 silencing, significantly reducing OSCC subcutaneous xenograft volume and weight, accompanied by a decrease in proliferation marker expression (Figure 2G–M; Figure S5M–R, Supporting Information). While the body weight of mice was not affected (Figure 2J). These findings demonstrated that reducing intracellular copper, either through SLC31A1 silencing or copper chelation, effectively suppresses OSCC cell proliferation in vitro and tumor growth in vivo.

Following the in vivo experimental results, we conducted an in‐depth scRNA‐seq analysis on subcutaneous xenograft tumors in C3H mice to elucidate the effects of targeting intracellular copper. This comprehensive approach, outlined in Figure S6A (Supporting Information), provided a granular view of cellular changes induced by the treatment. Using UMAP dimensionality reduction, we observed striking differences in clustering patterns between the control and TEPA‐treated groups (Figure S6B, Supporting Information). Then, these cells were subsequently classified into 23 clusters based on marker gene expression (Figure S6C, Supporting Information) and further categorized into seven major cell types including tumor epithelial, fibroblast, T/NK cell, monocyte, Neutrophil, Macrophage and DC (Figure 2N). Considering copper impacts on tumor cells, we mainly focused on the analysis of tumor epithelial cells. Upon isolating the tumor epithelial cell population, we further subdivided it into six clusters based on specific marker genes (Figure 2O). Notably, TEPA treatment led to a significant decrease in the proportion of cluster 0 and increases in clusters 3, 4, and 5 compared to the control group (Figure 2P). Marker genes for each cluster within the tumor epithelial population are shown in a heatmap (Figure S6D, Supporting Information), followed by functional enrichment analysis revealing associations with cell stemness (clusters 0, 1, 2), immune response (cluster 3), histone modification (cluster 4), and autophagy (cluster 5), indicating TEPA treatment decreased cell stemness subpopulation and increased immune response, histone modification, autophagy subpopulations in tumor epithelial populations (Figure S6E, Supporting Information).

These functional associations were further supported by CytoTRACE analysis, which revealed the highest stemness (differentiation potential) in clusters 0, 1, and 2 (Figure 2Q,R; Figure S7A,B, Supporting Information). Further validation using Multiplexed immunofluorescence demonstrated a significant reduction in stemness markers Cxcr4, Cd44, and Pdgfrb in TEPA‐treated tumors (Figure 2S; Figure S7C, Supporting Information).

### 2.3 Intratumor Copper is Required for EZH2 Protein Stability

**Figure 3:**
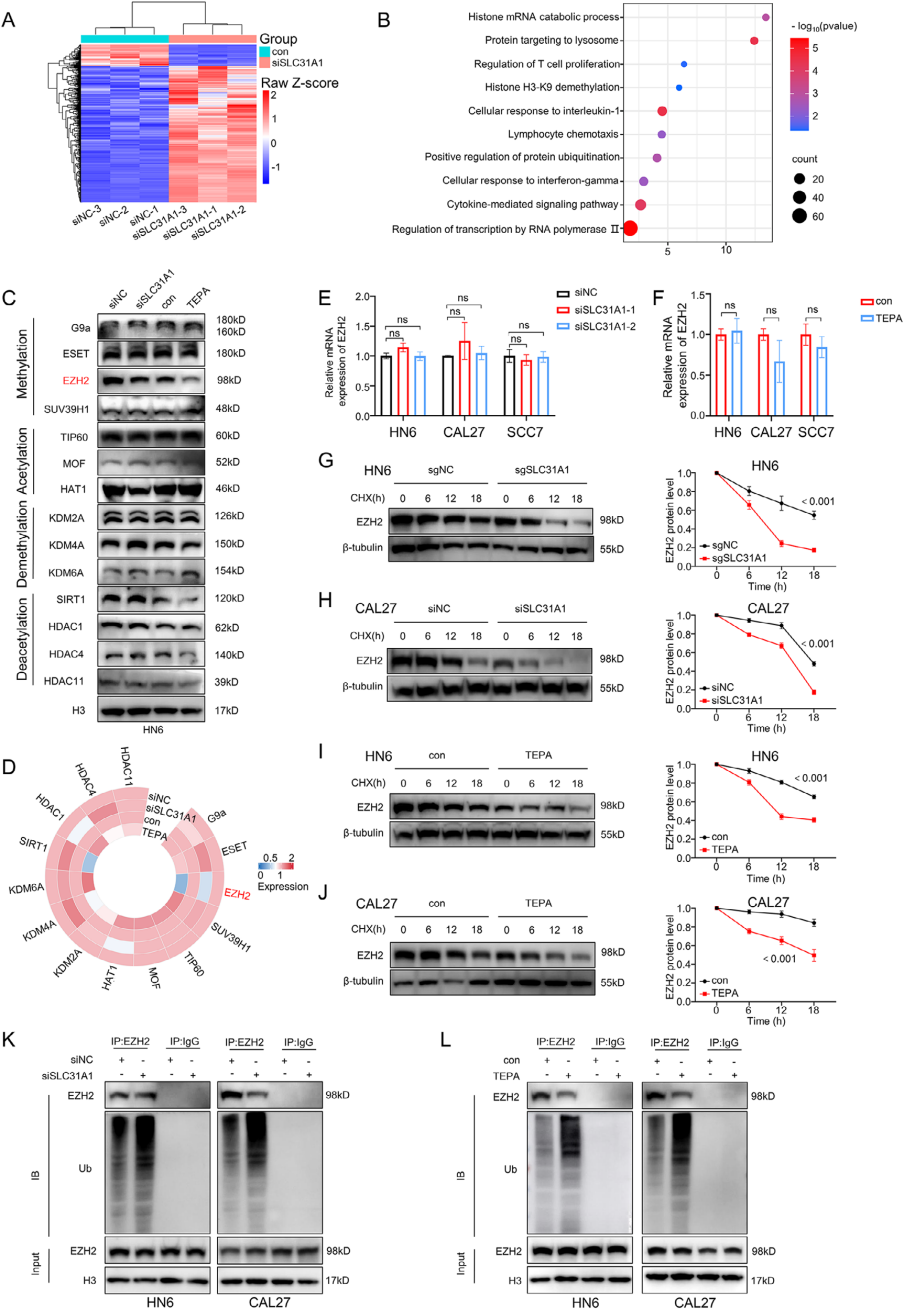
Intratumor copper is required for EZH2 protein stability. A) Differential gene expression analysis of HN6 cells with silenced SLC31A1 and control HN6 cells using RNA‐seq data. B) Pathway enrichment analysis of the differentially expressed genes. C,D) Detection of protein expression levels of histone‐modifying enzymes in HN6 cells after targeting intracellular copper. E) Changes in EZH2 mRNA levels under SLC31A1 silencing condition. F) Changes in EZH2 mRNA levels under treatment with copper ion chelator TEPA. G,H) Detection of EZH2 protein expression levels in SLC31A1‐KO HN6 (G) and SLC31A1‐silenced CAL27 (H) cells treated with CHX (20 µg mL^−1^) (0, 6, 12, and 18 h). I,J) Detection of EZH2 protein expression levels in TEPA‐treated HN6 (I) and CAL27 (J) cells treated with CHX (20 µg mL^−1^) (0, 6, 12, and 18 h). K,L) Ubiquitination immunoprecipitation experiments detecting changes in EZH2 ubiquitination levels in SLC31A1‐silenced (K) and TEPA‐treated (L) HN6 and CAL27 cells. Data in E‐J were calculated by two‐tailed unpaired Student's t test.

Having established the impact of targeting intracellular copper on cancer cell stemness, we sought to elucidate the underlying molecular mechanisms. We initiated our study by conducting RNA sequencing on control and SLC31A1‐silenced groups to globally map primary mediated by copper levels (**Figure** **3**A). This analysis revealed a significant enrichment of histone modification‐related pathways upon SLC31A1 silencing (Figure 3B), suggesting a potential link between copper and epigenetic regulation in the context of OSCC.

To validate this finding, we evaluate the protein level of each of the histone modification enzymes. We observed that targeting intracellular copper notably decreased the expression of the EZH2 protein (Figure 3C,D), a recognized regulator intimately linked to maintaining cell stemness. Our investigation also revealed a positive correlation between EZH2 and SLC31A1 expression in both OSCC cell lines and OSCCs. Significantly, concurrent high expression of both proteins was associated with poorer overall survival of OSCC patients (Figure S8, Supporting Information), underscoring the clinical relevance of this potential molecular interplay. Intriguingly, reducing intracellular copper significantly attenuated EZH2 protein levels, while its transcriptional expression was not altered (Figure 3E,F). This observation pointed toward a regulatory mechanism of EZH2 by copper that may operate at the translational or post‐translational level. To further explore this mechanism, we assessed the EZH2 protein half‐life, which demonstrated that decreasing intracellular copper accelerated EZH2 protein degradation (Figure 3G–J). Interestingly, we discovered that reducing intracellular copper enhanced the interaction between EZH2 protein and ubiquitin molecules (Figure 3K,L). This finding suggests that copper modulates EZH2 protein stability through the ubiquitination pathway. Collectively, these results delineate a mechanistic pathway linking intracellular copper levels to tumor cell stemness through the modulation of EZH2 protein stability.

### 2.4 Intracellular Copper Deficiency Promoted p62‐Mediated Selective Autophagic Degradation of EZH2

The ubiquitin‐proteasome and autophagy systems stand as the two most critical cellular degradation pathways, both utilizing ubiquitin as a common tag for substrate degradation.^[^ ^25^ ^]^ These pathways play pivotal roles in maintaining cellular homeostasis and normal physiological functions. Given their significance, we sought to elucidate the specific mechanism through which lowering intracellular copper levels leads to EZH2 protein degradation.

**Figure 4:**
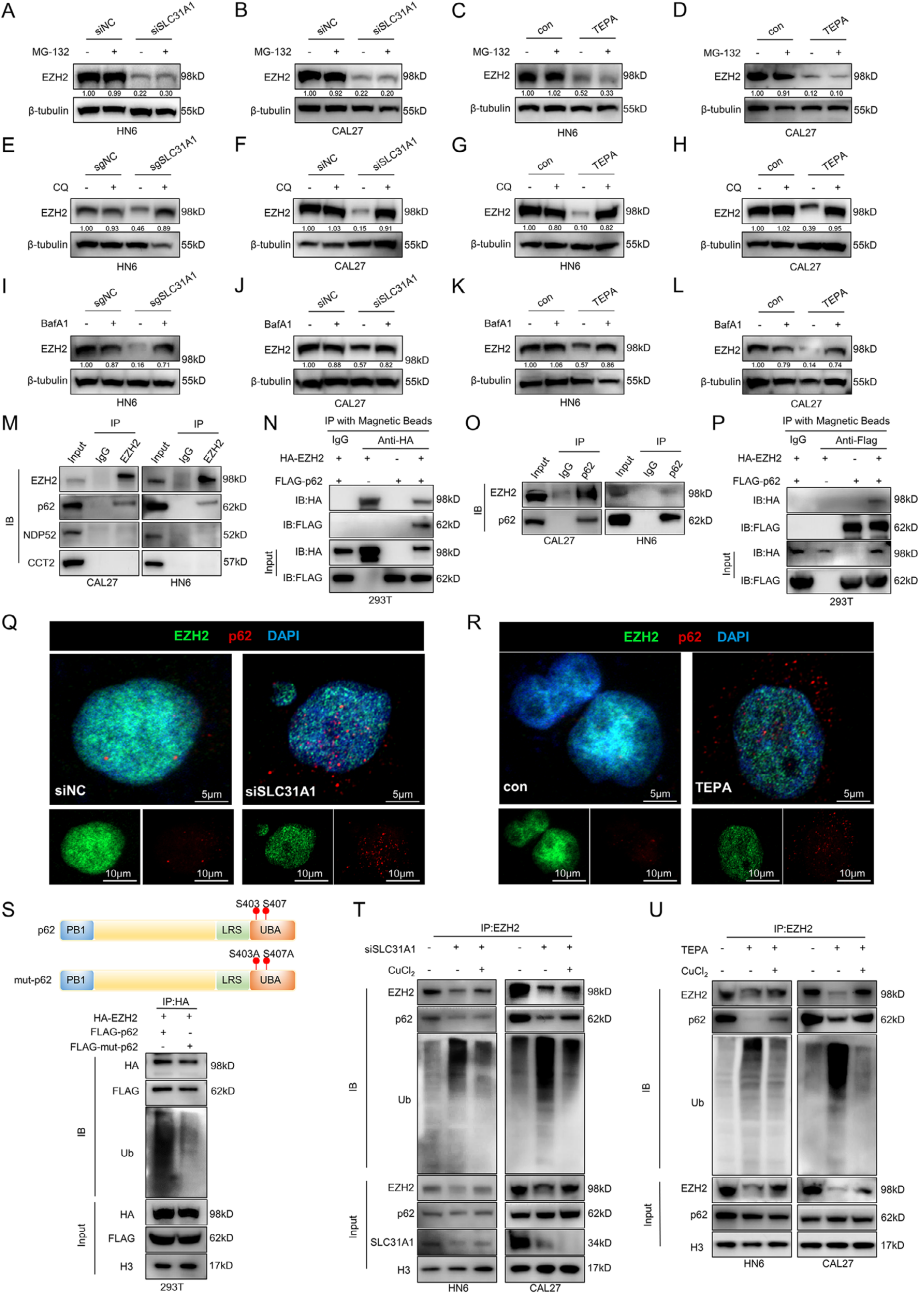
Intracellular copper deficiency promoted p62‐mediated selective autophagic degradation of EZH2. A,B) Detection of EZH2 protein expression differences with or without MG‐132 treatment in SLC31A1‐silenced HN6 (A) and CAL27 (B) cells. C,D) Detection of EZH2 protein expression differences with or without MG‐132 treatment in TEPA‐treated HN6 (C) and CAL27 (D) cells. E,F) Detection of EZH2 protein expression differences with or without CQ treatment in SLC31A1‐KO HN6 (E) and SLC31A1‐silenced CAL27 (F) cells. G,H) Detection of EZH2 protein expression differences with or without CQ treatment in TEPA‐treated HN6 (G) and CAL27 (H) cells. I,J) Detection of EZH2 protein expression differences with or without BafA1 treatment in SLC31A1‐KO HN6 (I) and SLC31A1‐silenced CAL27 (J) cells. K,L) Detection of EZH2 protein expression differences with or without BafA1 treatment in TEPA‐treated HN6 (K) and CAL27 (L) cells. M) Immunoprecipitation experiments were conducted to detect autophagy receptors interacting with EZH2 in HN6 and CAL27 cells. N) Overexpression of p62 and EZH2 alone or together in HEK293T cells, and immunoprecipitation experiments were performed to detect the interaction between EZH2 and p62. O) Immunoprecipitation experiments were carried out to detect the interaction between p62 and EZH2 in HN6 and CAL27 cells. P) Overexpression of p62 and EZH2 alone or together in HEK293T cells, and immunoprecipitation experiments were conducted to detect the interaction between p62 and EZH2. Q,R) Co‐localization experiments were performed to detect the co‐localization of EZH2 and p62 after targeting intracellular copper, Scale bars, 5 µm (top), 10 µm (bottom). S) Overexpression of EZH2 and p62 or mutant p62 in HEK293T cells, and immunoprecipitation experiments were conducted to detect the ubiquitination level of EZH2. T,U) Detection of EZH2 ubiquitination level in SLC31A1‐silenced (T) and TEPA‐treated (U) cells after rescuing trace amounts of copper ions by immunoprecipitation experiments in HN6 and CAL27 cells.

Initially, we explored the potential involvement of the proteasome pathway. Intriguingly, treatment with the proteasome inhibitor MG‐132 failed to restore EZH2 protein levels when intracellular copper was attenuated (**Figure** **4**A–D). This observation prompted us to investigate the autophagic degradation. Remarkably, the application of the autophagy inhibitor CQ, as well as Bafilomycin A1 (BafA1), resulted in a significant recovery of EZH2 expression (Figure 4E–L). This finding strongly indicated that the degradation of EZH2, induced by targeting intracellular copper, occurs predominantly through the autophagic degradation pathway. To further substantiate this conclusion, additional experiments were conducted and demonstrated that decreasing intracellular copper significantly elevated autophagy flux in OSCC cells (Figure S9, Supporting Information).

Selective autophagic degradation depends on specific cargo receptors to recognize ubiquitinated substrate.^[^ ^26^, ^27^ ^]^ To identify which cargo receptor is required for the autophagic degradation of EZH2, we next sought to identify the specific cargo receptor mediating EZH2 degradation. Through both endogenous and exogenous immunoprecipitation assays, we unequivocally identified cargo receptor p62 as the critical mediator in the degradation of the EZH2 protein (Figure 4M–P). This conclusion was further supported by colocalization experiments, which visually demonstrated the coexistence of EZH2 and p62 within the nuclei (Figure 4Q,R). The role of p62 in mediating ubiquitination and degradation of cargo proteins is well‐established, primarily through its UBA domain. To further elucidate this mechanism, we conducted site‐specific mutagenesis of the phosphorylation sites within the UBA domain (S403A, S407A) (Figure 4S). This mutation resulted in a notable reduction in the interaction between EZH2 and ubiquitin molecules, providing additional evidence for the p62‐mediated targeting of EZH2 for degradation in response to intracellular copper modulation. In our further experiments, we observed that reducing intracellular copper enhanced the interaction between EZH2 protein and ubiquitin molecules. Importantly, this interaction was significantly attenuated upon the restoration of trace amounts of copper. This observation provides compelling evidence that EZH2 protein stability is indeed regulated by intracellular copper levels (Figure 4T,U). Collectively, these findings delineate a novel mechanistic pathway linking intracellular copper levels to EZH2 protein stability via lysosomal‐mediated degradation.

### 2.5 Intracellular Copper Depletion Recruited and Stabilized E3 Ligase SMURF2 to Ubiquitinate EZH2

Building upon our initial observations of EZH2 and p62 colocalization within the nucleus, we embarked on a comprehensive investigation into the subcellular dynamics of copper‐induced EZH2 protein degradation.

**Figure 5:**
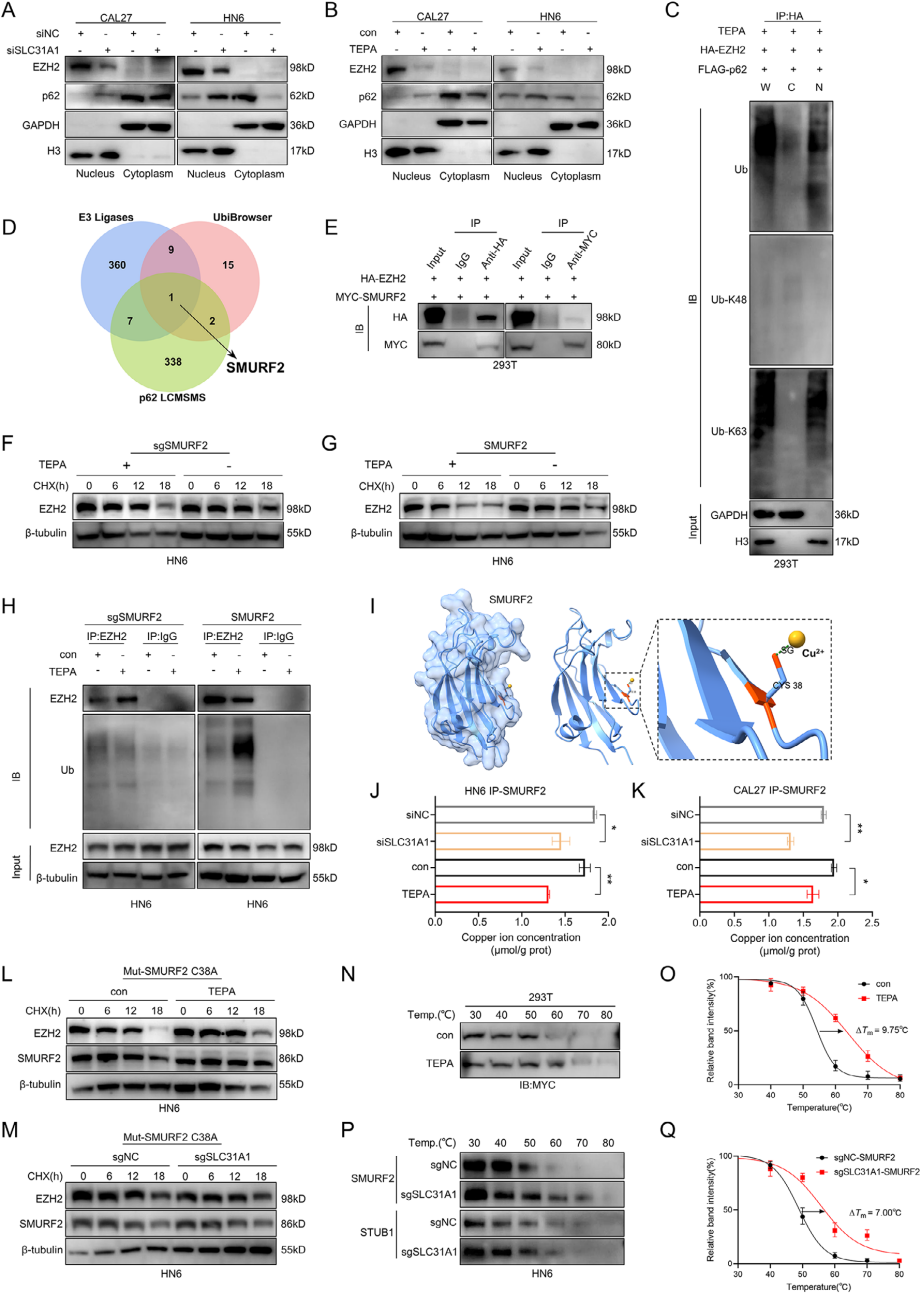
Intracellular copper depletion recruited and stabilized E3 ligase SMURF2 to ubiquitinate EZH2. A,B) Detection of EZH2 protein expression and localization in SLC31A1‐silenced (A) and TEPA‐treated (B) cells by nuclear‐cytoplasmic fractionation experiments in CAL27 and HN6 cells. C) Detection of EZH2 ubiquitination level by combining nuclear‐cytoplasmic fractionation and immunoprecipitation experiments in 293T cells co‐overexpressing EZH2 and p62 and treated with TEPA. D) Intersecting the datasets from E3 Ligases and UbiBrowser with mass spectrometry analysis results for p62 protein. E) Immunoprecipitation experiments were carried out to detect the interaction between SMURF2 and EZH2 in 293T cells. (F,G) Treated with or without TEPA and detected EZH2 protein expression levels in SMURF2‐KO (F) and SMURF2‐overexpression (G) HN6 cells treated with CHX (20 µg mL^−1^) (0, 6, 12, and 18 h). H) Treated with or without TEPA and detected the EZH2 ubiquitination level in SMURF2‐KO and SMURF2‐overexpression HN6 cells. I) Predict the binding site of SMURF2 with copper ions using AlphaFold 3. J) Detection of copper ion concentration in SLC31A1‐silenced and TEPA‐treated HN6 (ip‐SMURF2) cells. K) Detection of copper ion concentration in SLC31A1‐silenced and TEPA‐treated CAL27 (ip‐SMURF2) cells. L,M) Detection of EZH2 and SMURF2 protein expression levels in TEPA‐treated (L) and SLC31A1‐KO (M) HN6 cells treated with CHX (20 µg mL^−1^) (0, 6, 12, and 18 h) in the case of SMURF2 mutation. N,O) Detection of SMURF2 thermal stability treated with TEPA via Cellular Thermal Shift Assay (CETSA) in 293T cell. P,Q) Detection of SMURF2 and STUB1 thermal stability via CETSA in SLC31A1‐KO HN6 cell. Data in J‐K were calculated by two‐tailed unpaired Student's t test; Data in O and Q were calculated by nonlinear regression (curve fit).

Nuclear‐cytoplasmic fractionation assays revealed a decrease in EZH2 protein expression within the nucleus, concurrent with translocation of the p62 protein into the nucleus (**Figure** **5**A,B). This finding suggested a potential shift in the balance of EZH2 protein across cellular compartments. Further probing the nature of EZH2 interactions, we discovered a significant association between EZH2 and ubiquitin molecules within the nucleus. Notably, this interaction predominantly involved K63‐linked ubiquitination rather than K48‐linked ubiquitination (Figure 5C). This specific ubiquitination pattern is often associated with non‐degradative signaling, adding an intriguing layer of complexity to our understanding of EZH2 regulation. These results strongly indicate that the copper‐induced degradation of EZH2 is a nuclear event, challenging previous assumptions about the spatial organization of this process.

To elucidate the molecular machinery driving EZH2 degradation, we next aimed to identify the responsible E3 ubiquitin ligase by a multi‐faceted approach. By intersecting datasets from E3 Ligases (National Heart Lung and Blood Institute, NHLBI) and UbiBrowser with mass spectrometry analysis of p62 interacting protein following copper chelation, we identified SMURF2 as a candidate (Figure 5D). Subsequent co‐IP experiments validated the interaction between EZH2 and SMURF2 (Figure 5E), providing strong evidence for the role of SMURF2 in EZH2 degradation. We further generated SMURF2‐KO (sgSMURF2) (Figure S10A, Supporting Information) and SMURF2‐overexpressing HN6 cell lines. Under basal conditions, neither SMURF2 depletion nor overexpression significantly altered EZH2 protein stability or ubiquitination levels (Figure S10B–D, Supporting Information). However, TEPA treatment abolished the impact of SMURF2‐KO on EZH2 stability and ubiquitination, while SMURF2 overexpression markedly accelerated EZH2 ubiquitination‐mediated degradation (Figure 5F–H). These findings collectively demonstrate that SMURF2‐dependent EZH2 degradation is specifically induced under copper starvation conditions. To establish the relationship between intracellular copper and the function of SMURF2, we utilized AlphaFold 3 to predict a potential copper‐binding site (CYS 38) on SMURF2 (Figure 5I). This computational prediction was substantiated by co‐IP experiments, which demonstrated a significant reduction in copper ions interacting with SMURF2 upon copper chelation (Figure 5J,K). These findings suggest a direct interaction between SMURF2 and copper, which reduces its E3 ligase activity. Additionally, we measured SMURF2 mRNA levels (Figure S10E,F, Supporting Information), protein half‐life (Figure S10G,H, Supporting Information) and protein ubiquitination (Figure S10I, Supporting Information) in TEPA‐treated and SLC31A1 KO HN6 cells. The results indicated that intracellular copper depletion slightly reduced SMURF2 mRNA levels but had no statistically significant effect on SMURF2 protein half‐life or ubiquitination. Then we introduced a C38S mutation in SMURF2 to evaluate EZH2 protein half‐life (Figure 5L,M). The results demonstrated that SMURF2 mutations significantly attenuated TEPA‐induced effects on EZH2 protein stability. To further investigate the impact of intracellular copper on SMURF2 stability, we employed the Cellular Thermal Shift Assay (CETSA). This revealed that copper chelation increased the cellular thermal stability of SMURF2 (Figure 5N–Q; Figure S10J–M, Supporting Information), providing the possibility for a copper‐induced conformational change of SMURF2. We further investigated the relationship between SMURF2 and SLC31A1 expression in tumor tissues from 10 OSCC patients. Notably, no statistically significant correlation was detected between SMURF2 and SLC31A1 expression levels in these tumor samples (Figure S10N, Supporting Information). Based on these comprehensive findings, we propose a novel hypothesis that decreasing intracellular copper enhances SMURF2 activity through abolishing its inhibition by binding of copper, thereby promoting EZH2 protein degradation.

To elucidate the role of intracellular copper in OSCC progression, we further investigated its impact on the activity of the EZH2‐associated PRC2 complex. Initially, we evaluated the transcriptional regulation of three well‐established EZH2 target genes: DAB2IP, RUNX2, and CDKN1A. Then, utilizing EZH2 ChIP‐qPCR, we examined the binding activity of EZH2 on the promoter of these genes upon CAL27 cells treated with siSLC31A1 or TEPA (Figure S11A,B, Supporting Information). Our findings revealed a striking correlation between reduced intracellular copper levels and decreased EZH2 binding to its target genes. This observation strongly suggests that copper deficiency impairs the function of the PRC2 complex, a key regulator of gene expression and cellular identity.

To further investigate the functional consequences of this copper‐EZH2 interplay, we conducted a set of rescue experiments. Following SLC31A1 silencing, which reduces intracellular copper levels, we overexpressed EZH2 via plasmid transfection. Remarkably, this intervention led to a significant restoration of tumor cell proliferation and clone formation abilities (Figure S11C,D, Supporting Information). These in vitro findings provided compelling evidence for the EZH2‐dependent nature of copper's role in OSCC cell proliferation. To validate and extend these observations to a more physiologically relevant context, we conducted in vivo experiments using a subcutaneous xenograft model in mice. The results were striking: the growth capacity of the xenografts (Figure S11E–G, Supporting Information) and the expression of the proliferation marker Ki67 (Figure S11H, Supporting Information) were both substantially rescued upon restoration of EZH2 expression. This corroborated copper‐EZH2 axis is critical for OSCC progression. Collectively, our results provide robust evidence that reducing intracellular copper inhibits OSCC cell proliferation in an EZH2‐dependent manner. This conclusion is supported by a comprehensive array of experimental approaches, encompassing both in vitro and in vivo models.

### 2.6 Targeting Intracellular Copper Enhances the Therapeutic Efficacy of Anti‐PD‐1 Therapy in OSCC

**Figure 6:**
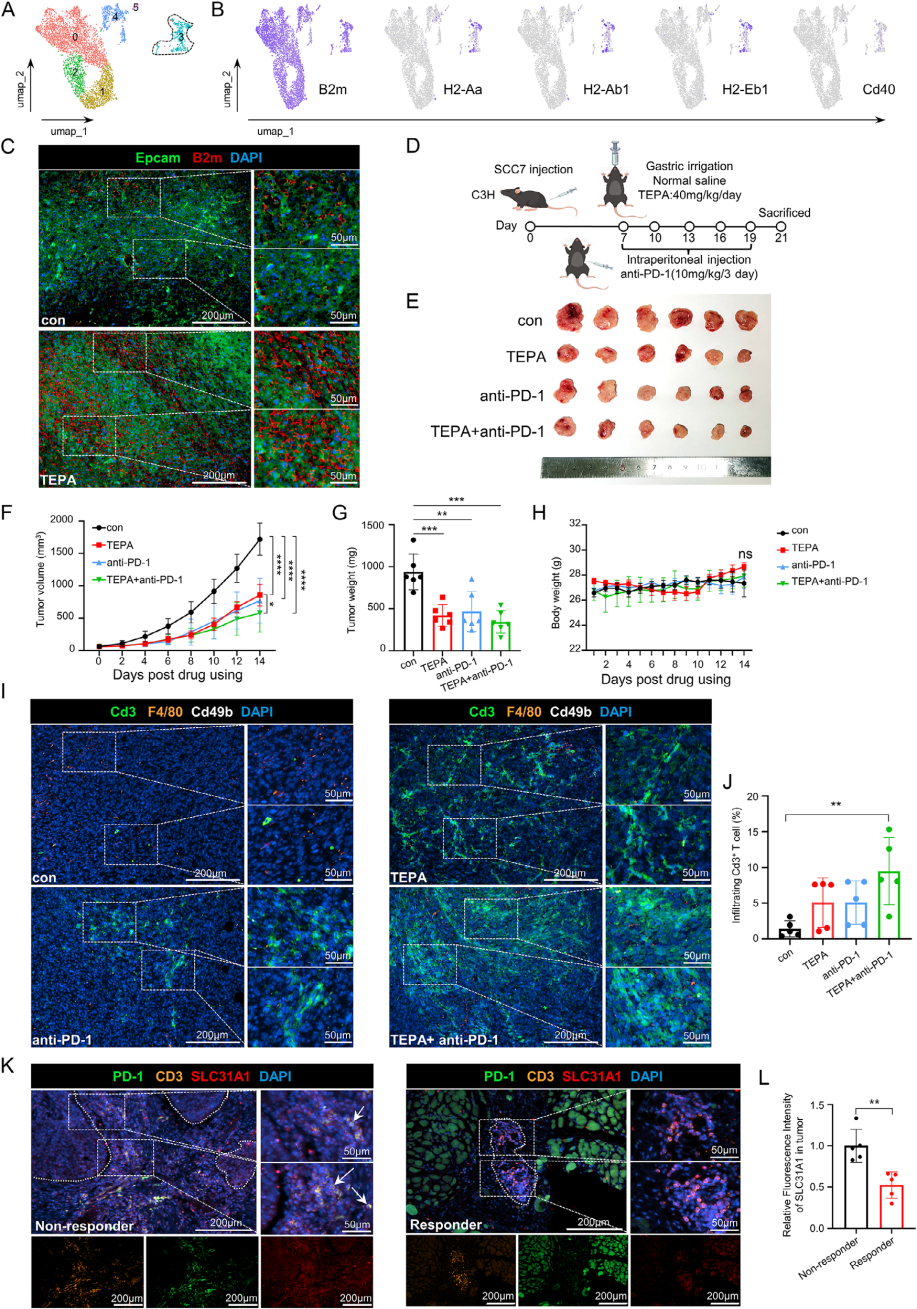
Targeting intracellular copper enhances the therapeutic efficacy of anti‐PD‐1 therapy in OSCC. A,B) The expression level of B2m, H2‐Aa, H2‐Ab1, H2‐Eb1 and Cd40 in tumor epithelial cell in C3H mice. B) Multiplexed immunofluorescence of Epcam and B2m in subcutaneous xenograft tissues from C3H mice. D) Schematic diagram of subcutaneous tumor (SCC7) formation in C3H mice, TEPA oral administration, and PD‐1 monoclonal antibody intraperitoneal injection experiment. E) Macroscopic view of subcutaneous transplanted tumors in C3H mice. F) Changes in volume of subcutaneous transplanted tumors in C3H mice. G) Weight of subcutaneous transplanted tumors in C3H mice. H) Body weight of C3H mice. I,J) Multiplexed immunofluorescence was performed on paraffin sections of transplant tumor tissues from C3H mice to detect Cd3, F4/80 and Cd49b. K,L) Through Multiplexed immunofluorescence, the relationship between SLC31A1 expression and immune response is evaluated in OSCC patients who are resistant or sensitive to immunotherapy. The area outlined by the white dotted line represents the tumor tissue. Data in F‐H, J, and L were calculated by two‐tailed unpaired Student's t test.

Anti‐PD‐1 therapy was approved by the FDA in the treatment of recurrent and metastatic HNSCC patients.^[^ ^21^, ^22^ ^]^ However, the low ORR of anti‐PD‐1 therapy alone in patients with HNSCC indicates a significant number of patients do not experience clinical benefits, suggesting that combined treatment strategies may improve clinical benefits. Our scRNA‐seq analysis revealed an intriguing increase in tumor cell immunogenicity following treatment with the copper chelator TEPA (**Figure** **6**A–C). Additionally, we stratified TCGA‐OSCC data into EZH2‐high and EZH2‐low expression groups and conducted immune infiltration analysis. Notably, the EZH2‐low group exhibited significantly higher dendritic cell (DC) enrichment scores compared to the EZH2‐high group (Figure S12A, Supporting Information), suggesting that reduced EZH2 expression may enhance DC‐mediated antigen presentation and antitumor immunity. These prompted further investigation into the potential of targeting intracellular copper to enhance tumor immune response and improve immunotherapy efficacy. Specifically, our experimental results demonstrated a significant reduction in the growth capacity of OSCC subcutaneous xenograft tumors in C3H mice when treated with the copper chelator TEPA combined with anti‐PD‐1 therapy (Figure 6D–G). Importantly, this potent anti‐tumor effect was achieved without significant impact on the mice's body weight (Figure 6H). Further analysis revealed a marked increase in T‐cell infiltration within the tumor microenvironment following the combination treatment, accompanied by a concomitant decrease in macrophage infiltration (Figure 6I,J). Further investigation demonstrated that the combination treatment significantly reduced the abundance of M2‐polarized tumor‐associated macrophages (Figure S13, Supporting Information). This enhanced immune cell presence suggests a shift toward a more immunologically active tumor milieu, potentially explaining the improved therapeutic efficacy.

Extending our findings to human OSCC patients, we observed that tumors exhibiting lower SLC31A1 expression, indicative of potential sensitivity to immunotherapy, displayed significantly higher T‐cell infiltration and reduced T‐cell exhaustion phenotypes compared to resistant tumors, indicating a noteworthy correlation between SLC31A1 expression levels and immunotherapy responsiveness (Figure 6K,L). This clinical data supported our experimental findings and highlights the potential translational relevance of targeting intracellular copper as a strategy to enhance immunotherapy efficacy.

**Figure 7:**
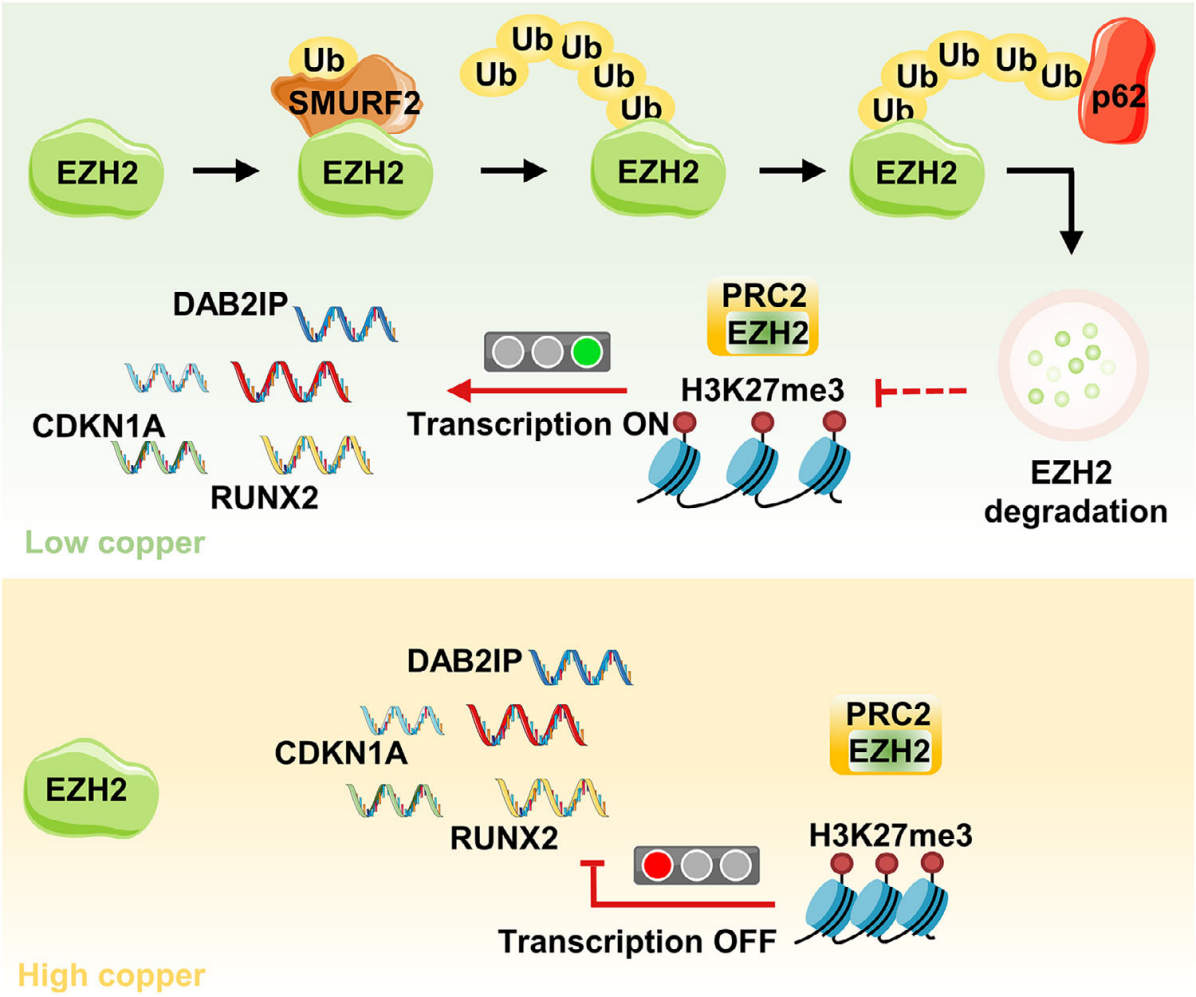
Schematic diagram showing the regulatory mechanisms of p62‐mediated EZH2 degradation. Copper modulates p62 binds to EZH2, and targeting intracellular copper reduces the binding of copper to SMURF2, thereby stabilizing SMURF2 and promoting p62‐mediated EZH2 ubiquitination and degradation within the nucleus.

Collectively, we uncovered a novel mechanism whereby copper chelation leads to p62‐mediated ubiquitination and degradation of EZH2 within the nucleus. This process appears to play a crucial role in modulating the tumor immune microenvironment, ultimately potentiating the efficacy of anti‐PD‐1 therapy (**Figure** **7**).

## 3 Discussion

Copper, an essential trace element, plays a pivotal role in both normal physiological processes and pathological conditions, including cancer.^[^ ^28^ ^]^ The regulation of intracellular copper levels is tightly controlled by specific transmembrane proteins responsible for its uptake and export in mammalian cells. A comprehensive pan‐cancer analysis has revealed that mutations, aberrant methylation, and overexpression of SLC31A1, a key player in copper uptake, are prevalent across various cancer types.^[^ ^11^ ^]^ Our study is devoted to the investigation of copper homeostasis in OSCC, demonstrating elevated copper levels in the serum of OSCC patients compared to healthy individuals. These elevated copper levels correlate with clinical stage, tumor size, and lymph node metastasis in OSCC patients, accompanied by upregulation of SLC31A1. This pattern extends beyond OSCC, as we observed similar trends in breast, esophageal, and lung cancers across two independent cohorts. Notably, dysregulation of SLC31A1 showed a strong association with advanced clinical stage, increased tumor size, and poor overall survival.

The concept of cuproplasia, where copper directly facilitates cancer initiation and progression, has gained traction in recent years. Copper dysregulation emerges as a critical oncogenic factor across multiple malignancies. In lung cancer, cuproptosis—a copper‐dependent cell death mechanism—has been implicated in therapeutic responses, with copper chelators demonstrating potential to modulate tumor metabolism and sensitivity to targeted therapies.^[^ ^29^ ^]^ Similarly, in melanoma, elevated copper levels correlate with aggressive tumor biology, yet copper depletion via chelators attenuates proliferation and overcomes drug resistance, underscoring its dual regulatory role.^[^ ^30^ ^]^ Prostate cancer studies further reveal that copper imbalance drives tumor progression, with compounds like complanatoside A inducing cuproptosis by disrupting copper homeostasis, offering novel therapeutic avenues.^[^ ^31^ ^]^ Collectively, these findings highlight copper metabolism as a actionable target in oncology. Copper exerts its oncogenic effects through multiple mechanisms, including activation of the mitogen‐activated protein kinase (MAPK) and oncogenic BRAF pathways, promoting tumor cell proliferation.^[^ ^32^ ^]^ Furthermore, copper influences the activity of metalloenzymes such as lysyl oxidase (LOX),^[^ ^33^ ^]^ enhancing tumor cell metastasis. And activating fibroblast growth factors (FGFs), vascular endothelial growth factor (VEGF), and pro‐inflammatory cytokines like interleukin (IL)‐1, IL‐6, and IL‐8, promoting angiogenesis.^[^ ^34^ ^]^ Preclinical and clinical evidence supports copper chelation as a viable anticancer strategy. In breast cancer, TTM demonstrated therapeutic potential by disrupting copper‐dependent pathways, reducing tumor proliferation, and improving survival outcomes.^[^ ^35^ ^]^ Mechanistic studies reveal that copper chelators induce immunogenic cell death through cuproptosis regulation, with agents like D‐penicillamine and trientine (TETA4) showing synergy with immunotherapy in melanoma models.^[^ ^36^ ^]^ Notably, TETA4's established safety profile in Wilson's disease underscores its translational potential for repurposing in oncology.^[^ ^37^ ^]^ These collective findings validate copper metabolism as an actionable therapeutic target across multiple malignancies, warranting expanded clinical evaluation. Our study employed two distinct strategies to target intracellular copper: copper chelators and SLC31A1 knockdown. Both approaches yielded significant reductions in OSCC cell proliferation and tumor growth, both in vitro and in vivo. Mechanistically, we uncovered that targeting intracellular copper modulates the protein levels of EZH2 and the activity of the EZH2‐associated PRC2 complex. Silencing SLC31A1 or treatment with the copper chelator TEPA reduced EZH2 binding to its target genes, indicating compromised PRC2 complex function under copper‐deficient conditions. The functional significance of this EZH2 modulation was further confirmed through EZH2 rescue experiments. In HN6 and CAL27 cells, EZH2 overexpression markedly reversed the anti‐proliferative and colony formation‐inhibiting effects of SLC31A1 knockdown. In vivo, EZH2 overexpression significantly increased tumor volumes and weights in xenografts derived from HN6 cells with SLC31A1 knockdown, correlating with increased expression of the proliferation marker Ki67. These findings strongly suggest that copper deficiency reduces OSCC cell proliferation in an EZH2‐dependent manner.

Wilson's disease is a hereditary disorder of copper metabolism, characterized by toxic accumulation of copper in the liver, brain, and other organs. Long‐term safety data for copper chelators in Wilson's disease (WD) management provide critical insights for therapeutic optimization. A retrospective analysis of 43 WD patients demonstrated sustained tolerability of trientine tetrahydrochloride over 12.6‐year median exposure, with minimal hepatic adverse events.^[^ ^38^ ^]^ Similarly, a multicenter trial confirmed comparable efficacy and safety profiles between teta4 and penicillamine, favoring TETA4 for its favorable metabolic profile.^[^ ^37^ ^]^ Notably, innovative formulations like pH‐responsive chelator carriers^[^ ^39^ ^]^ demonstrate potential to reduce off‐target effects, while zinc co‐therapy balances efficacy with neuroprotective benefits.^[^ ^40^ ^]^ Although TTM showed reduced intestinal copper absorption in healthy volunteers,^[^ ^41^ ^]^ its clinical utility requires further validation. These studies collectively support the established long‐term safety of copper‐targeted therapies in WD, with ongoing pharmacovigilance essential to refine therapeutic indices. For the safety assessment of copper starvation treatment, we performed hematoxylin‐eosin (HE) staining on the liver, spleen, lungs, and kidneys of mice. The histopathological analysis revealed no significant morphological changes in these organs following copper starvation treatment (Figure S12C,D, Supporting Information). Additionally, serum biochemical assays for liver and kidney function markers (Figure S12E, Supporting Information) showed no abnormalities, indicating that acute copper depletion does not impair hepatorenal function in this model. However, chronic toxicity evaluations and extended pharmacovigilance are required to assess potential long‐term adverse effects of copper‐targeted therapies in future studies.

Our previous research has established EZH2 as an oncogenic driver in the malignant transformation of oral leukoplakia and as an independent prognostic predictor for HNSCC patients.^[^ ^42^, ^43^ ^]^ We have also elucidated mechanisms by which EZH2 expression is regulated in OSCC, including the role of lncRNA RC3H2 in sponging miR‐101‐3p to release EZH2 expression^[^ ^44^ ^]^ and the action of lncRNA DUXAP9 in blocking CDK1‐mediated EZH2 degradation.^[^ ^45^ ^]^ The present study unveils a novel mechanism that EZH2 undergoes degradation upon copper depletion. We demonstrate that copper mediates a p62‐associated EZH2 Ub‐K63 ubiquitination and degradation in the nucleus via an autophagy‐dependent, rather than ubiquitin proteasome‐dependent pathway. Autophagy is an evolutionarily conserved mechanism, characterized by a sequence of tightly orchestrated steps that enable the degradation and recycling of cellular constituents. This process is integral to the regulation of various cellular activities, including inflammation, infection, metabolic balance, and tumor development.^[^ ^46^ ^]^ Recent findings underscore the essential role of selective autophagy, which utilizes specialized cargo receptors to target ubiquitinated substrates, in the precise modulation of cancer dynamics.^[^ ^27^ ^]^ For example, NOD‐like receptor 6 (NLRP6) enhances the interaction between the cargo receptor optineurin and p85α, encoded by phosphoinositide‐3‐kinase regulatory subunit 1 (PIK3R1), thereby amplifying PI3K/AKT signaling to drive tumorigenesis. Conversely, disrupting the NLRP6‐p85α interaction has been shown to suppress tumor progression.^[^ ^47^ ^]^ Our findings provide new insights into the modulation of EZH2 degradation in cancer cells and expand the understanding of the intricate relationship between cellular metabolism and epigenetic regulation.

The clinical implications of our findings extend to immunotherapy. While anti‐PD‐1 and anti‐PD‐L1 therapies have shown promise in recurrent and metastatic HNSCC,^[^ ^2^, ^3^ ^]^ improving overall response rates remains a critical challenge. Combined therapeutic modalities with anti‐PD‐1 have been widely evaluated in preclinical and clinical trials.^[^ ^48^, ^49^, ^50^ ^]^ Copper has emerged as a potential regulatory factor in tumor immunity, with several studies elucidating its complex role in modulating immune responses within the tumor microenvironment.^[^ ^51^, ^52^, ^53^ ^]^ For example, in murine mesothelioma models, copper depletion has been associated with increased CD4+ T cell infiltration, suggesting a link between copper levels and immune cell recruitment.^[^ ^54^ ^]^ Additionally, the anti‐tumor agent Dp44mT has been shown to affect T cell activity through copper‐related mechanisms, highlighting copper's influence on T cell function.^[^ ^55^, ^56^ ^]^ In another study, selective copper chelation demonstrated efficacy in a drug‐resistant tumor model by promoting apoptosis in bone marrow‐derived suppressor cells (MDSCs), thereby enhancing anti‐tumor immune responses.^[^ ^57^ ^]^ In lung cancer, downregulation of ACO3, a copper‐containing amine oxidase, has been associated with reduced adherent aggregation of CD4+ T cells.^[^ ^58^ ^]^ Moreover, a phase II study on high‐risk and triple‐negative breast cancer patients found that TTM decreased collagen deposition, lowered MDSCs levels, and promoted CD4+ T cell infiltration in treated mice.^[^ ^59^ ^]^ In addition, copper ions might regulate the expression of PD‐L1, suggesting a potential role for copper ions in tumor therapy by modulating immune checkpoints.^[^ ^28^ ^]^ Our study conducted a preliminary assessment of combination therapy efficacy at day 14 (Figure 6D), though ethical considerations related to tumor burden necessitated early humane endpoints and tissue harvesting at this timepoint. This represents a critical limitation of our experimental design, as long‐term therapeutic dynamics could not be evaluated. Future investigations will employ orthotopic models with slower tumor progression kinetics to enable extended efficacy monitoring and survival analysis, while incorporating serial biopsies to track dynamic changes in copper metabolism and immune microenvironment remodeling during prolonged treatment.

Our work identifies copper metabolism as a therapeutic vulnerability in OSCC, with direct translational implications. By targeting SLC31A1‐mediated copper influx and EZH2‐driven immunosuppression, our findings support repurposing copper chelators (e.g., TEPA) or developing SLC31A1 inhibitors to disrupt tumor‐intrinsic copper addiction. Clinical validation could transform treatment paradigms for aggressive OSCC subsets, particularly when combined with immunotherapy. Biomarker‐driven trials using copper/EZH2 signatures may enhance precision oncology approaches. While anti‐PD‐L1 therapies (e.g., pembrolizumab, nivolumab) have improved outcomes in head and neck cancers, resistance mechanisms remain prevalent. Our strategy combining copper chelation with immunotherapy offers a distinct mechanism‐based approach: by reducing intratumoral copper to suppress EZH2‐mediated immunosuppression, we observe enhanced T cell infiltration and MHC‐I presentation in OSCC models. Unlike direct checkpoint inhibition, this approach targets metabolic reprogramming of tumor cells, potentially overcoming adaptive resistance. Future head‐to‐head trials should compare durability of response and biomarker‐driven patient selection between these modalities.

## 4 Conclusion

Taken together, our study elucidates a novel mechanism by which targeting cuproplasia suppresses the malignant phenotypes of OSCC cells. We have uncovered a previously unknown regulatory pathway wherein copper modulates p62 binds to EZH2, and targeting intracellular copper reduces the binding of copper to SMURF2, thereby stabilizing SMURF2 and promoting p62‐mediated EZH2 Ub‐K63 ubiquitination and degradation. Further research is warranted to fully characterize this pathway and to explore its potential as a therapeutic target in other cancer types. Additionally, clinical studies will be necessary to translate these findings into effective treatment options for OSCC patients.

## 5 Experimental Section

### Cell Lines and Cell Culture

CAL27 (RRID: CVCL_1107), HN6 (RRID: CVCL_8129), SCC7 (RRID: CVCL_V412), HEK293T (RRID: CVCL_0063), and HOK (RRID: CVCL_B405) were used in this study. HN6 cells were kindly provided by the University of Maryland Dental School, USA. The CAL27, SCC7, HEK293T, and HOK cell lines were obtained from the ATCC (American Type Culture Collection). CAL27, HN6, HEK293T, and HOK cells were cultured in Dulbecco's modified Eagle's medium (DMEM; BasalMedia, CN). The media were supplemented with 10% heat‐inactivated fetal bovine serum (FBS) (BasalMedia), penicillin (100 units mL^−1^), and streptomycin (100 µg mL^−1^). The cells were cultured at 37 °C in a humidified 5% CO_2_ atmosphere.

### Chemicals

Tetraethylenepentamine pentahydrochloride (TEPA, 375 683), Tetrathiomolybdate (TTM, 323 446), copper chloride hydrate (CuCl_2_, C3279), MG‐132 (proteasome inhibitor, M7449), Chloroquine (CQ, autophagy inhibitor, C6628) were purchased from Sigma‐Aldrich (Shanghai) Trading Co., Ltd.

### Animal Study

C3H/He mice and nude mice were purchased from Shanghai Bikai Keyi Biotechnology Co., Ltd. All the mice were bred and housed in the animal facility of Shanghai Ninth People's Hospital under specific pathogen‐free conditions. All animal experiments were randomized and conducted in accordance with the Guide for the Care and Use of Laboratory Animals. All the animal experiments were approved by the Laboratory Animal Ethics Committee in Ninth People's Hospital, Affiliated with Shanghai Jiao Tong University School of Medicine. Animal experiments implemented in BALB/C nude mice (6 weeks old) (Shanghai Laboratory Animal Center, Shanghai, China), were conducted in accordance with the appropriate ethical standards and national guidelines. siNC, siSLC31A1, Vector or EZH2 cells (5 × 10^6^) were suspended in 0.1 mL of sterile PBS and subcutaneously injected into the left dorsal of mice. Animal experiments implemented in C3H mice (6 weeks old) (Shanghai Laboratory Animal Center, Shanghai, China), were conducted in accordance with the appropriate ethical standards and national guidelines. siNC, siSLC31A1 cells (5 × 10^5^) were suspended in 0.1 mL of sterile PBS and subcutaneously injected into the left and right dorsal flanks of mice. The intragastric administration dose of TEPA was 40 mg kg^−1^ day^−1^, and the intraperitoneal injection dose of the anti‐PD‐1 was 10 mg kg^−1^ every 3 days. Tumor volumes (length × width^2^ / 2) were monitored and compared.

### Detection of Metal Ion Concentration

Fresh blood samples were centrifugated at 15,000 g for 10 min, after which the supernatant (serum) was collected. The concentration of copper ions in the serum was subsequently determined using a Serum Copper Content Detection Kit (Solarbio, BC5640). For the cell samples, the harvested cells were homogenized with 0.15 mL of double‐distilled water per 2 × 10^6^ cells. After centrifugation, the supernatant was obtained, and the copper ion concentration was measured using a Cell Copper Colorimetric Assay Kit (Elabscience, E‐BC‐K775‐M); the calcium ion concentration was measured using a Cell Copper Colorimetric Assay Kit (Solarbio, BC8333); the zinc ion concentration was measured using a Cell Copper Colorimetric Assay Kit (Solarbio, BC2810); the iron ion concentration was measured using a Cell Copper Colorimetric Assay Kit (Solarbio, BC5310); the magnesium ion concentration was measured using a Cell Copper Colorimetric Assay Kit (Solarbio, BC8335).

### siRNA or Plasmid Transfection

The siRNA used in the study was designed and synthesized by Guangzhou RiboBio Co., Ltd. (Guangzhou, China). The plasmid was designed and synthesized by Genomeditech (Shanghai, China). Transfection was performed using Lipofectamine 3000 reagent (Invitrogen) following the manufacturer's instructions. The sgRNA sequence of Human SLC31A1: sgRNA1: 5′‐TGGATCATTCCCACCATATG‐3′; sgRNA2: 5′‐AGAAGGTTGCATGGTACTGT‐3′; sgRNA3: 5′‐GTGATGGTGAGAAGGTTGCA‐3′. The sgRNA sequence of Human SMURF2: sgRNA1: 5′‐TCTGCCAGGGCTAGAATATT‐3′; sgRNA2: 5′‐GTGGACAGTCTTCAGATCCC‐3′; sgRNA3: 5′‐ACAGTCTTCAGATCCCAGGC‐3′.

### RNA Extraction and qRT‐PCR

Total RNA from tissues and cells was extracted using TRIzol reagent (Invitrogen, USA) and used to generate cDNA with HiScript II Q RT SuperMix (Vazyme, CN). Real‐time PCR was performed using a LightCycler 480 RealTime PCR System (Roche, USA) with 2x SYBR Green qPCR Master Mix (Bimake, USA) according to the manufacturer's instructions. The PCR primers were designed and synthesized by Sangon Biotech (Shanghai) Co., Ltd. and are listed in Table S1 (Supporting Information).

### Cell Counting Kit‐8 (CCK‐8) Analysis

Cells transfected for 24 h with siRNA/sgRNA or plasmid were seeded into 96‐well plates at a density of 1000 cells per well in triplicate. The cells were harvested, and 10 µL of CCK‐8 reagent (Dojindo, Kumamoto, Japan) was added to 100 µL of culture medium. The cells were subsequently incubated for 2 h at 37 °C, and the optical density was measured at 450 nm using a microplate reader (Multiskan Sky Spectrophotometer, Thermo Scientific, USA).

### Colony‐Forming Assay

Cells transfected for 24 h with siRNA/sgRNA or plasmid were seeded into 6‐well plates at a density of 1000 cells per well and incubated for 10–14 days to form cell colonies. The colonies were fixed with 4% paraformaldehyde (Wuhan Servicebio Technology Co., Ltd., China), stained with 1% crystal violet (Biosharp, China), and then counted under a dissecting microscope.

### Chromatin Immunoprecipitation (ChIP)

According to the manufacturer's instructions, ChIP assays were performed on CAL27 cells with a SimpleChIP Enzymatic Chromatin IP Kit (#S9003, CST, USA). IgG was used as the negative control, and anti‐EZH2 antibody (#5246, CST, USA, RRID: AB_10 694 683) was used to pull down the promoter regions of target genes. Then, ChIP DNA was analyzed by qPCR using specific primers, and the data were normalized to the input DNA. The results were derived from three independent experiments. The primers used for ChIP‐qPCR are listed in Table S2 (Supporting Information).

### Coimmunoprecipitation (co‐IP)

For the co‐IP assay, cells were lysed with IP buffer (150 mm NaCl, 50 mm Tris‐HCl pH = 8.0, 1% Nonidet P‐40, 25 mm NaF, 2 mm Na3VO4, and 1% protease inhibitor cocktails and 5 mm PMSF before use) on ice for 30 min, and the cell lysate was harvested by centrifugation. The precleaning step was followed by incubation with the indicated antibodies and Protein A/G magnetic beads (B23202, Bimake, USA) at 4 °C overnight. The next day, the beads bound by target proteins were washed 6 times with IP buffer. Proteins were denatured at 95 °C for 10 min for WB analysis.

### Ubiquitination Assay

Cultured cells were treated with 20 µm MG132 (HY‐13259, MCE, USA) for 6 h and then lysed in IP lysis buffer containing protease and phosphatase inhibitors on ice for 30 min. Anti‐EZH2 antibody (#5246, CST, dilution 1:100, RRID: AB_10 694 683) or IgG was added to the lysate and incubated with rotation overnight at 4 °C. Protein A/G magnetic beads (B23202, Bimake, USA) were added to the mixture, incubated at 4 °C for 2 h, boiled in SDS loading buffer, and used for western blotting analysis. Antiubiquitin antibody (#3936, CST, dilution 1:1000, RRID: AB_331 292) or anti‐HA antibody (51064‐2‐AP, Proteintech, USA, RRID: AB_11 042 321) was used to detect the ubiquitination of EZH2.

### Western Blot Analysis

Cells were collected at the indicated times in SDS lysis buffer (Beyotime, China). The protein concentration was determined by a BCA protein assay kit (Beyotime, China). Equal amounts of proteins were separated by sodium dodecyl sulfate‐polyacrylamide gel electrophoresis (SDS‐PAGE) and transferred to polyvinylidene difluoride membranes. The membrane was soaked in 10% skim milk in PBS for 1 h at room temperature and incubated with primary antibody overnight at 4 °C. Then, secondary antibodies were used. Afterward, the protein‐antibody complex was visualized by enhanced chemiluminescence assay (SB‐WB012, Share‐bio, China). 𝛽‐tubulin, 𝛽‐actin, GAPDH, and H3 were used as control. Antibodies against SLC31A1 (NB100‐402, Novus Biologicals, USA, dilution 1:1000, RRID: AB_10 003 309), 𝛽‐tubulin (#2128, CST, USA, dilution 1:1000, RRID: AB_823 664), 𝛽‐actin (66009‐1‐Ig, Proteintech, USA, dilution 1:50 000, RRID: AB_2 687 938), ERK1/2 (11257‐1‐AP, Proteintech, USA, dilution 1:1000, RRID: AB_2 139 822), Phospho‐ERK1/2 (Thr202/Tyr204) (28733‐1‐AP, Proteintech, USA, dilution 1:1000, RRID: AB_2 881 202), G9a (#3306, CST, USA, dilution 1:1000, RRID: AB_2 097 647), ESET (66293‐1‐Ig, Proteintech, USA, dilution 1:1000, RRID: AB_2 881 676), EZH2 (#5246, CST, USA, dilution 1:1000, RRID: AB_10 694 683), SUV39H1 (#8729, CST, USA, dilution 1:1000, RRID: AB_10 829 612), TIP60 (10827‐1‐AP, Proteintech, USA, dilution 1:1000, RRID: AB_2 128 431), MOF (13842‐1‐AP, Proteintech, USA, dilution 1:1000, RRID: AB_2 146 894), HAT1 (67971‐1‐Ig, Proteintech, USA, dilution 1:1000, RRID: AB_2 918 721), KDM2A (24311‐1‐AP, Proteintech, USA, dilution 1:1000, RRID: AB_2 879 488), KDM4A (29977‐1‐AP, Proteintech, USA, dilution 1:1000, RRID: AB_2 923 625), KDM6A (23984‐1‐AP, Proteintech, USA, dilution 1:1000, RRID: AB_2 935 460), SIRT1 (60303‐1‐Ig, Proteintech, USA, dilution 1:1000, RRID: AB_2 881 417), HDAC1 (#5356, CST, USA, dilution 1:1000, RRID: AB_10 612 242), HDAC4 (#7628, CST, USA, dilution 1:1000, RRID: AB_10 860 255), HDAC11 (67949‐1‐lg, Proteintech, USA, dilution 1:1000, RRID: AB_2 918 701), H3 (BS1174, Bioworld, China, dilution 1:1000, RRID: AB_1 663 967), H3K27me3 (#9733, CST, USA, dilution 1:1000, RRID: AB_2 616 029), mTOR Pathway Antibody Sampler Kit (#9964, CST, USA, RRID: AB_10 696 892), FLAG (20543‐1‐AP, Proteintech, USA, dilution 1:1000, RRID: AB_11 232 216), HA (51064‐2‐AP, Proteintech, USA, dilution 1:1000, RRID: AB_11 042 321), p62 (T59081, Abmart, China, dilution 1:1500, RRID: AB_2 936 470), NDP52 (A7358, Abclonal, China, dilution 1:1000, RRID: AB_2 767 894), CCT2 (A4700, Abclonal, China, dilution 1:1000, RRID: AB_2 863 327), LC3B (T55992, Abmart, China, dilution 1:1000, RRID: AB_2 929 010), GAPDH (A19056, Abclonal, China, dilution 1:1000, RRID: AB_2 862 549), K48‐linkage Specific Polyubiquitin(#8081, CST, USA, dilution 1:1000, RRID: AB_10 859 893), K63‐linkage Specific Polyubiquitin(#5621, CST, USA, dilution 1:1000, RRID: AB_10 827 985), MYC (60003‐2‐Ig, Proteintech, USA, dilution 1:2000, RRID: AB_2 734 122), STUB1 (55430‐1‐AP, Proteintech, USA, dilution 1:1000, RRID: AB_10 949 225) and SMURF2 (18038‐1‐AP, Proteintech, USA, dilution 1:1000, RRID: AB_3 085 549) were used.

### Immunohistochemistry (IHC)

Paraffin‐embedded tissue sections were dewaxed and rehydrated before antigen retrieval by boiling in 10 mm citrate buffer (pH = 6.0) for 30 min. Then, 3% hydrogen peroxide was added for 15 min to remove endogenous peroxidase. Tissues were incubated with goat serum for 30 min at room temperature and then with anti‐SLC31A1 (NB100‐402, Novus Biologicals, USA, dilution 1:100, RRID: AB_10 003 309), anti‐EZH2 (#5246, CST, USA, dilution 1:100, RRID: AB_10 694 683) antibodies at 4 °C overnight. Immunodetection was performed on the following day using DAB (Servicebio) according to the manufacturer's instructions. The staining scores were determined by two independent observers based on both the proportion and brown intensity of the indicated protein‐positive cells.

### Multiplexed Immunofluorescence

Dewaxing and antigen epitope retrieval of paraffin sections of OSCC samples and mouse‐bearing tumors were processed as mentioned in IHC. The permeabilization process for tissue sections was conducted with 0.5% v/v TritonX‐100 in PBST, and Fc‐blocking was conducted with 10% v/v goat serum in PBST. Interaction of primary antibodies and fluorescence‐conjugated secondary antibodies was conducted following general procedure: Sections were incubated with EZH2 (#5246, CST, USA, dilution 1:100, RRID: AB_10 694 683), Ki67 (#GB111499, Servicebio, China, dilution 1:200, RRID: AB_2 927 572), SLC31A1 (NB100‐402, Novus Biologicals, USA, dilution 1:100, RRID: AB_10 003 309), EPCAM (21050‐1‐AP, Proteintech, USA, dilution 1:200, RRID: AB_10 693 684), CXCR4 (60042‐1‐lg, Proteintech, USA, dilution 1:500, RRID: AB_2 091 809), CD44 (60224‐1‐lg, Proteintech, USA, dilution 1:500, RRID: AB_11 042 767), PDGFRB (#3169, CST, USA, dilution 1:200, RRID: AB_2 162 497), CD3 (17617‐1‐AP, Proteintech, USA, dilution 1:200, RRID: AB_1 939 430), PD‐1 (#86 163, CST, USA, dilution 1:300, RRID: AB_2 728 833), F4/80 (#30 325, CST, USA, dilution 1:500, RRID: AB_2 798 990), CD49b (A19068, Abclonal, China, dilution 1:100, RRID: AB_2 862 560), CD163 (16646‐1‐AP, Proteintech, USA, dilution 1:300, RRID: AB_2 756 528) in 4 °C, overnight and washed with PBST three times before incubated with fluorescence‐conjugated secondary antibodies at room temperature, in the dark, for 2 h, and unconjugated secondary antibodies were removed by PBST before another primary antibody incubation. For nuclei staining, tissue sections were incubated at room temperature with Hoechst 33 342 (Absin, abs813337) diluted in PBST at 1 µg mL^−1^ for 15 min. All immunofluorescence‐staining slides were mounted with Fluoromount‐G (Yeasen, 36307ES25). Fluorescent microscopy image capture and scanning were conducted with FV3000 (Olympus), and images were processed with Imaris (Oxford Instruments).

### Single‐Cell RNA‐Sequencing (scRNA‐seq) and Analysis

The fresh tissue sample was temporarily placed in RPMI‐1640 medium (Corning) with 20% fetal bovine serum (FBS, Cell Technologies) on ice and processed for scRNA‐seq following previously described protocols. scRNA‐seq libraries were prepared using Chromium TM Single Cell G Chip (10 × Genomics, 1 000 120) and Chromium Single Cell 3′ Library & Gel Bead Kit v3.1 (10 × Genomics, 1 000 121), and sequencing was accomplished on an Illumina NovaSeq6000 System using a paired‐end 150 bp. Sequenced reads were aligned and quantified using the Cell Ranger 5.0.0 pipeline. After applying these QC criteria, 12 158 high‐quality cells (300 < nFeature_RNA) were included in downstream analyses. The filtered gene expression matrix for each sample was normalized and scaled by the “NormalizeData” and “ScaleData” functions in Seurat. Principal component analysis (PCA) was performed on the corrected expression matrix using highly variable genes (HVGs) identified by the “FindVariableGenes” function. Next, the “RunPCA” function was used to perform the PCA, and the “FindNeighbors” function was used to construct a K‐nearest‐neighbor graph. The most representative principal components were used to determine different cell types with the “FindCluster” function. “FindAllMarkers” (multiple condition comparisons) and “FindMarkers” (two condition comparisons) from the Seurat package were used with default parameters. The expression differences with P < 0.05 and log2 (fold change, FC) > 0.25 were considered as differentially expressed genes. CytoTRACE was also a scoring method that estimates developmental potential based on included datasets. To perform CytoTRACE (version 0.3.3), the default recommended settings were used after concatenating relevant batches through an outer join.

### Spheroid Formation Assay

Spheroid formation assays were performed to evaluate the regulation of cancer stem‐like cells (CSCs). OSCC cells were dissociated and suspended in a spheroid medium consisting of serum‐free DMEM/F12 (Gibco, USA), human bFGF (20 ng ml^−1^, Sino Biological Inc., China), human EGF (20 ng ml^−1^, Sino Biological Inc., China), and B‐27 supplement (Life Technologies, USA) in 6‐well ultra‐low attachment dishes (Corning, USA) under different treatment for 10 days. The spheroid morphology was observed microscopically, and the number of spheroids was counted and compared.

### RNA Sequencing and Analysis

RNA‐seq‐based transcriptome profiling was performed for the OSCC cells under different treatments by the Beijing Genomics Institute (Wuhan) using the BGISEQ‐500 platform. After filtering, the sequencing data were mapped to the human reference genome hg38 (Assembly: GCF_0 00001405.38_GRCh38.p12) using HISAT and Bowtie2 software. The transcript quantification and normalization were performed using the RSEM software package. The differentially expressed genes (DEGs) were identified by the R package (Q‐value ≤ 0.001 and fold change > 1.5). The DEGs were subjected to functional classification using GO and KEGG analysis.

### Immunocytochemistry

Cells for immunocytochemistry were processed similarly to those in the immunofluorescence assay by first fixing them in 4% paraformaldehyde for 15 min and then permeabilizing with 0.5% Triton‐X‐100 for 15 min. The samples were blocked and incubated in anti‐EZH2 (#5246, CST, USA, dilution 1:100, RRID: AB_10 694 683), anti‐p62 (T59081, Abmart, China, dilution 1:1500, RRID: AB_2 936 470). After incubation with Alexa Fluro anti‐mouse, anti‐rabbit, the samples were counterstained with DAPI and imaged with a Zeiss LSM780 confocal microscope.

### Cellular Thermal Shift Assay (CETSA)

HEK293T cells expressing SMURF2‐MYC and HN6 cells were treated with TEPA for 24 h or SLC31A1 silencing for 48 h and harvested. Equal volumes of cell suspensions from control and treated groups were aliquoted into PCR strip tubes. The heating procedure was carried out in a PCR Cycler (BioRad) with a gradient temperature program (30 to 80 °C) for 3 min, followed by immediate cooling on ice. After three freeze‐thaw cycles using liquid nitrogen, samples were centrifuged at 15000 × g for 30 min at 4 °C to pellet the denatured protein precipitation. The soluble SMURF2 proteins in the supernatants were analyzed by following Western blot analysis using anti‐MYC antibody (60003‐2‐Ig, Proteintech, USA, dilution 1:2000, RRID: AB_2 734 122), STUB1 (55430‐1‐AP, Proteintech, USA, dilution 1:1000, RRID: AB_10 949 225) and SMURF2 (18038‐1‐AP, Proteintech, USA, dilution 1:1000, RRID: AB_3 085 549).

### Statistics Analysis

Statistical analysis was performed using R (3.6.1). Statistical differences were evaluated using two‐tailed unpaired Student's t‐test for comparisons between two groups. A p value of less than 0.05 (*p < 0.05, **p < 0.01, ***p < 0.001, and ****p < 0.0001) was considered statistically significant. Statistical methods and corresponding p values for data shown in each panel were included in the figure legends. When comparing 3 or more groups, 1‐way ANOVA was used with Dunnett's (referring to 1 control group) or Tukey's (comparing several groups) analysis. For survival analyses, log‐rank analyses were performed.

### Ethics Approval and Consent to Participate

All blood samples were collected from the Department of Oral and Maxillofacial‐Head and Neck Oncology, Ninth People's Hospital, Shanghai Jiao Tong University School of Medicine. OSCC tissues were obtained from the Department of Oral and Maxillofacial‐Head and Neck Oncology, Ninth People's Hospital, Shanghai Jiao Tong University School of Medicine (2018‐86‐T77) and the Department of Oral and Maxillofacial‐Head and Neck Oncology, Beijing Stomatological Hospital, Capital Medical University (CMUSH‐IRB‐KJ‐PJ‐2021‐04). The responders and non‐responders after adjuvant immunotherapy were enrolled in this study from the Tianjin Medical University Cancer Institute and Hospital (Ek2020212). Informed consent was obtained from all patients. Written informed consents were received from all patients. All the animal experiments have been approved by the Laboratory Animal Ethics Committee in Ninth People's Hospital, Affiliated with Shanghai Jiao Tong University School of Medicine (SH9H‐2024‐A164‐SB). Ethical approvals from all participating institutions have been successfully obtained.

## Conflict of Interest

The authors declare no conflict of interest.

## Author Contributions

X.L., W.C., and B.L. contributed equally to this work. W.C. and C.L. designed and oversaw the project and revised the manuscript. X.L., W.C., and W.C. performed experiments and wrote the manuscript. Z.F., B.L., and Z.Z. analyzed the data and interpreted the results. X.Z., Z.Y., and Z.F. conducted single‐cell sequencing, and X.‐Y.Z., Z.F., and X.Z. revised the manuscript. All authors read and approved the manuscript.

## Supporting information



Supporting Information

## Data Availability

The datasets used and/or analyzed during the current study are available from the corresponding author on reasonable request.
