# Supplementary material for: Targeting Intratumoral Copper Inhibits Tumor Progression via p62‐Mediated EZH2 Degradation and Potentiates Anti‐PD‐1 Immunotherapy in Oral Squamous Cell Carcinoma
Source: Adv Sci (Weinh). 2025 Jul 28;12(40):e17795. doi: 10.1002/advs.202417795 (PMC12561351; doi:10.1002/advs.202417795)
Supplement: Supplementary file 1 — Supporting Information [file ADVS-12-e17795-s001.pdf]

Figure S1

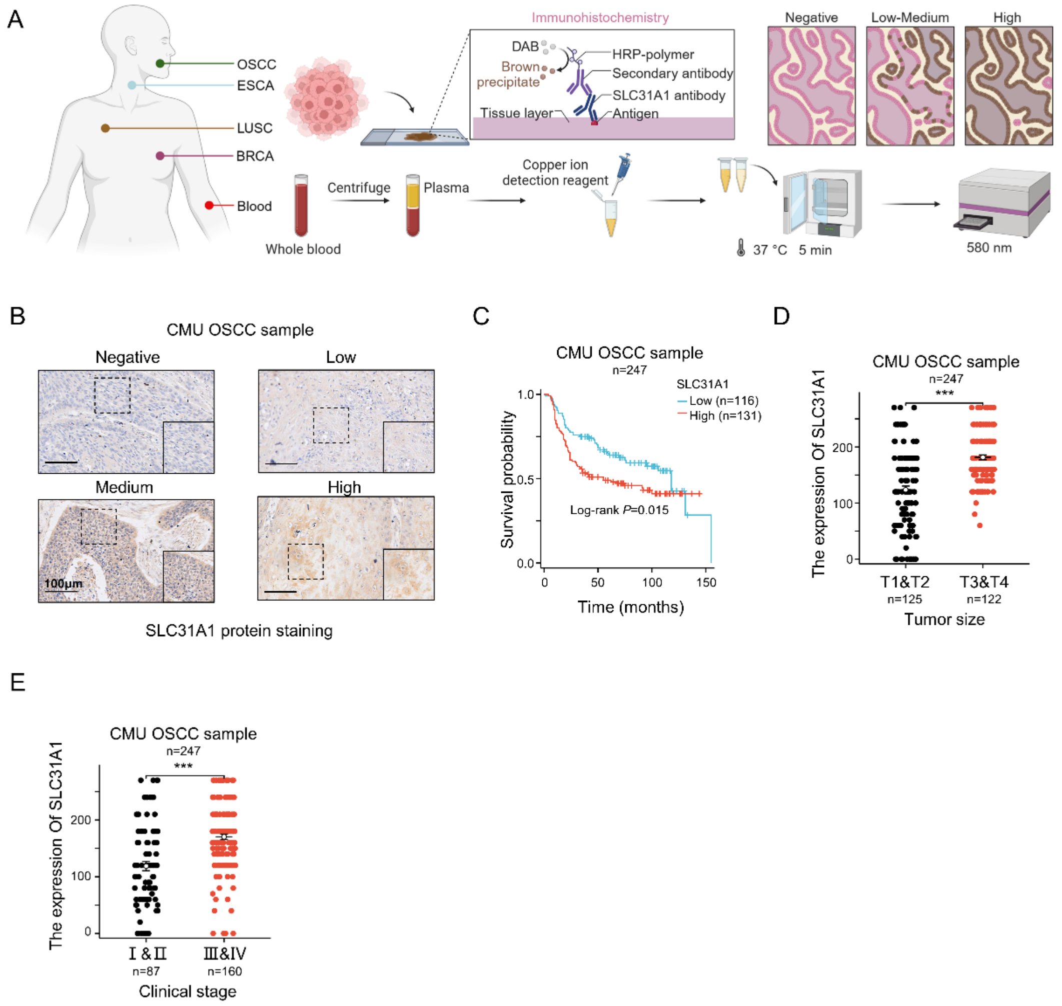

Figure S1. (**A**) Flowchart of serum and tumor sample processing. (**B-E**) In an independent clinical sample of OSCC from the Stomatology Hospital of Capital Medical University (CMU) (n=247) (**B**), the correlation between SLC31A1 expression and tumor clinical staging (**E**), tumor size (**D**), and patient prognosis (**C**) were analyzed, Scale bars, 100 μm. Data in D-E were calculated by two-tailed unpaired Student's t test. Data in C was analyzed by Kaplan–Meier plots, p values were determined by a two-tailed log-rank test.

Figure S2

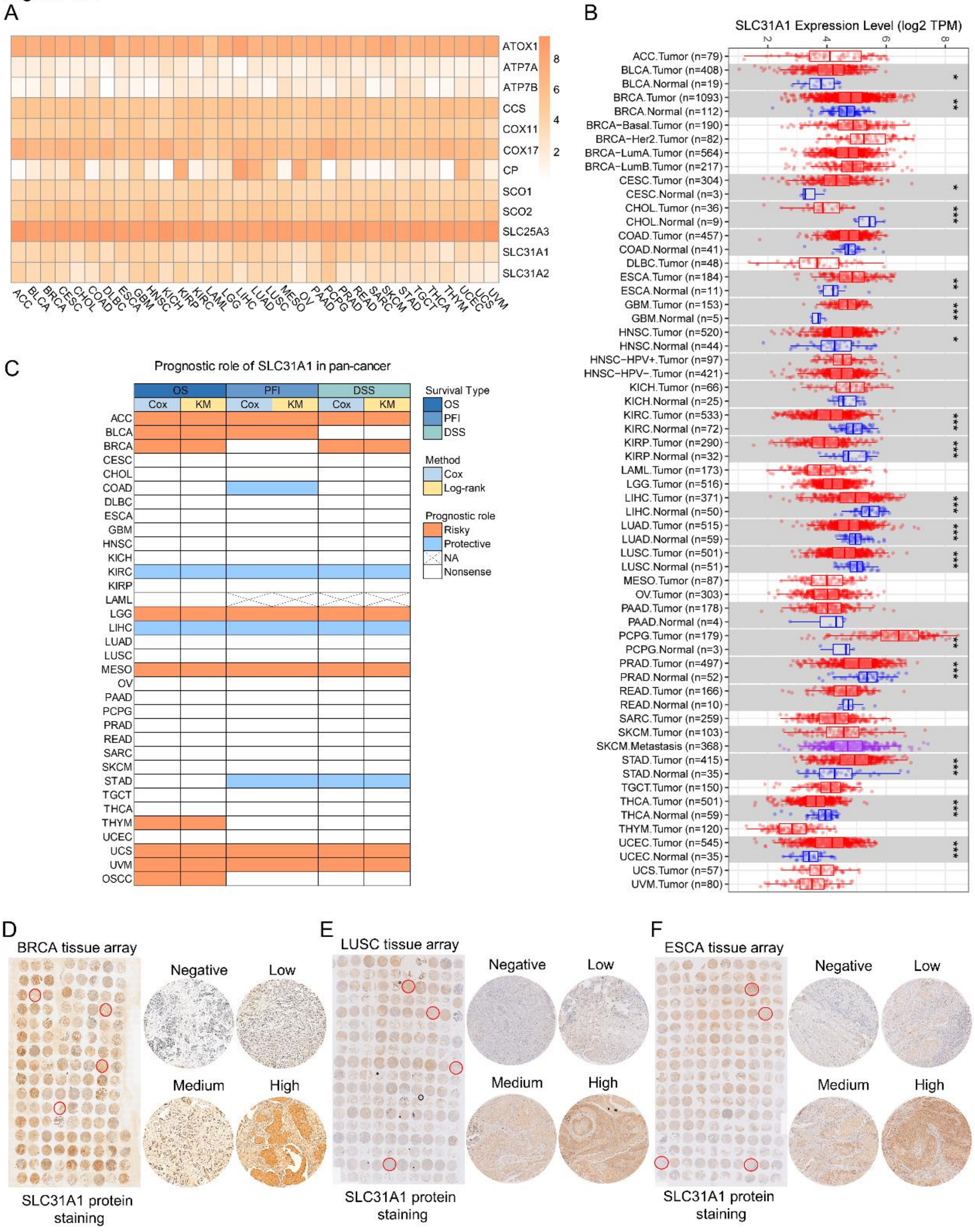

Figure S2. (**A**) Expression levels of copper transporter proteins in various types of cancer. (**B**) Expression levels of SLC31A1 in various types of cancer. (**C**) The prognostic significance of SLC31A1 in various types of cancer. (**D**) Expression levels of SLC31A1 in BRCA tissue microarrays (n = 132). (**E**) Expression levels of SLC31A1 in LUSC tissue microarrays (n = 85). (**F**) Expression levels of SLC31A1 in ESCA tissue microarrays (n = 112).

Figure S3

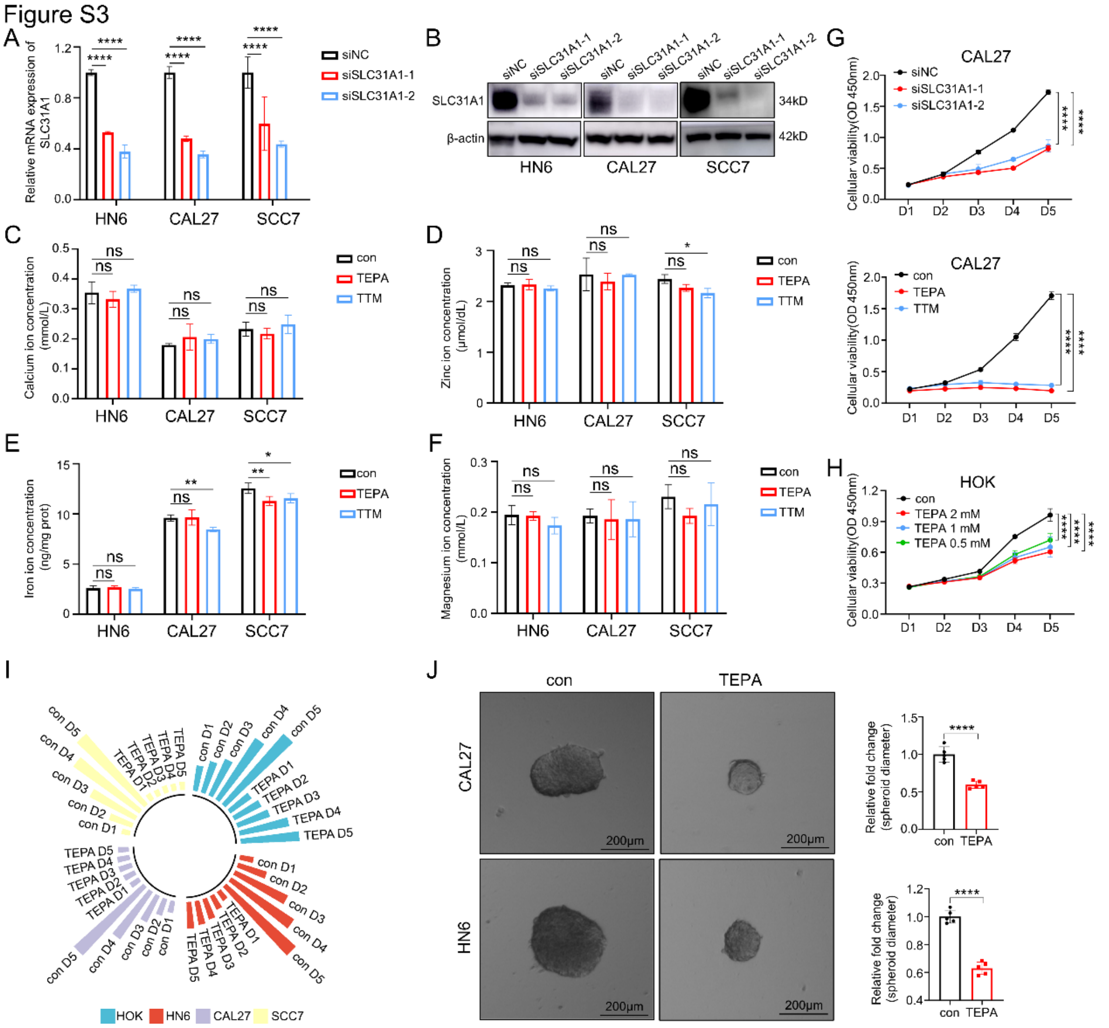

Figure S3. (**A**) Detection of mRNA expression levels in OSCC cells after silencing SLC31A1. (**B**) Detection of protein expression levels in OSCC cells after silencing SLC31A1. (**C**-**F**) TEPA and TTM were used in OSCC cell lines (HN6, CAL27 and SCC7) to measure changes in the concentrations of calcium ions (**C**), zinc ions (**D**), iron ions (**E**) and magnesium ions (**F**) within the cells. (**G**) The effect of silencing SLC31A1 and copper ion chelators on cell proliferation ability in CAL27. (**H**) The effect of TEPA on cell proliferation ability in human oral keratinocyte cells (HOK). (**I**) The effect of TEPA on proliferation ability of cells in OSCC cell lines and HOK. (**J**) The effect of copper ion chelators on sphere formation (sphere diameter) in OSCC cell lines, Scale bars, 200 μm. Data were calculated by two-tailed unpaired Student’s t test.

Figure S4

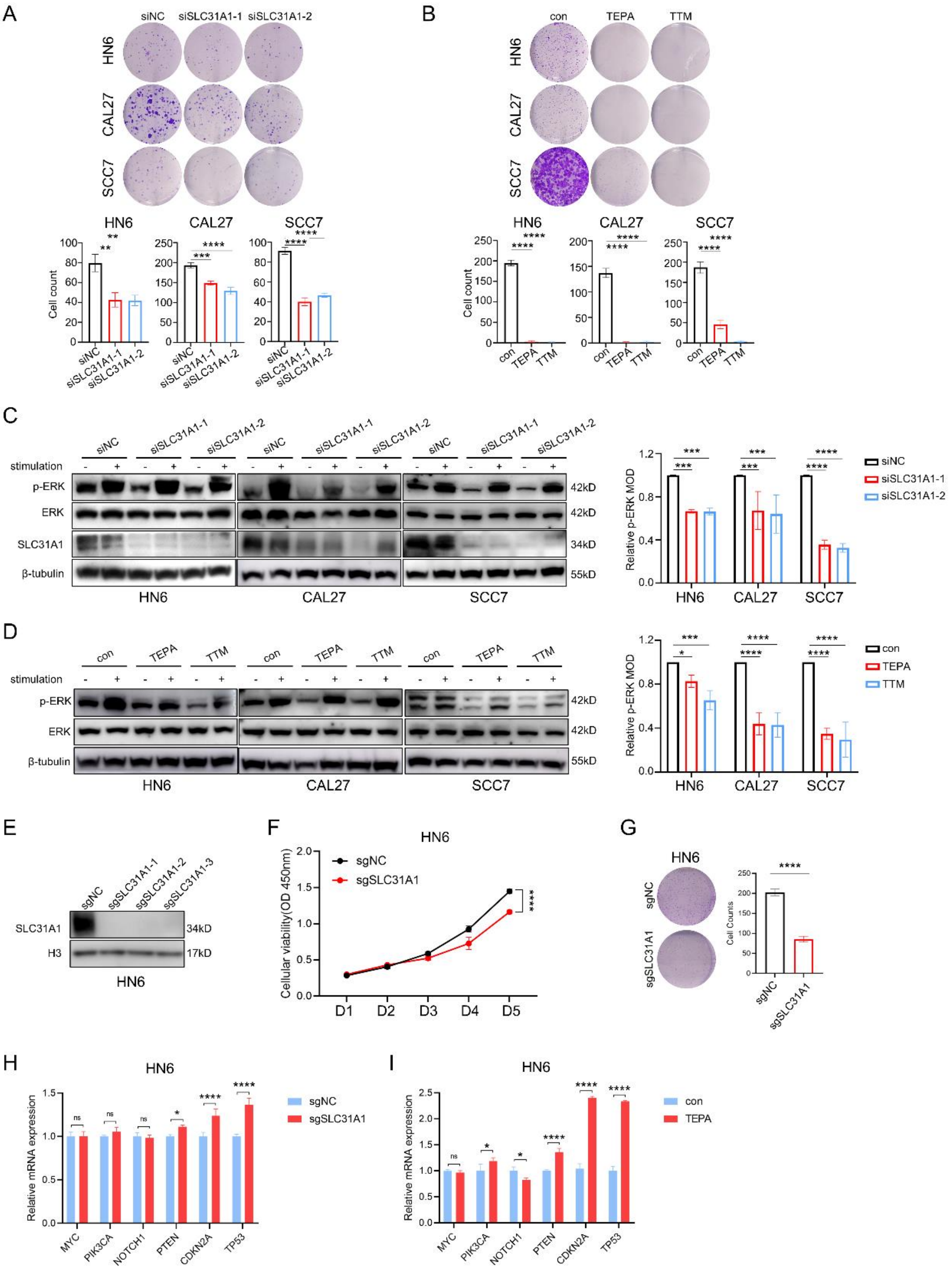

Figure S4. (**A**-**B**) The effect of silencing SLC31A1 (**A**) and copper ion chelators (**B**) on colony formation ability of cells in OSCC cell lines (HN6, CAL27 and SCC7). (**C**-**D**) The effect of silencing SLC31A1 (**C**) and copper ion chelators (**D**) on the expression levels of p-ERK protein in cells in OSCC cell lines (HN6, CAL27 and SCC7). (**E**) Detection of SLC31A1 protein

expression levels in HN6 cells after SLC31A1-KO. (**F**) The effect of SLC31A1-KO cell proliferation ability in HN6. (**G**) The effect of SLC31A1-KO on colony formation ability of cells in HN6. (**H**) The expression level of MYC, PIK3CA, NOTCH1, PTEN, CDKN2A and TP53 in SLC31A1-KO HN6 cells. (**I**) The expression level of MYC, PIK3CA, NOTCH1, PTEN, CDKN2A and TP53 in TEPA-treated HN6 cells. Data in A-D, F-**I** were calculated by two-tailed unpaired Student's t test.

Figure S5

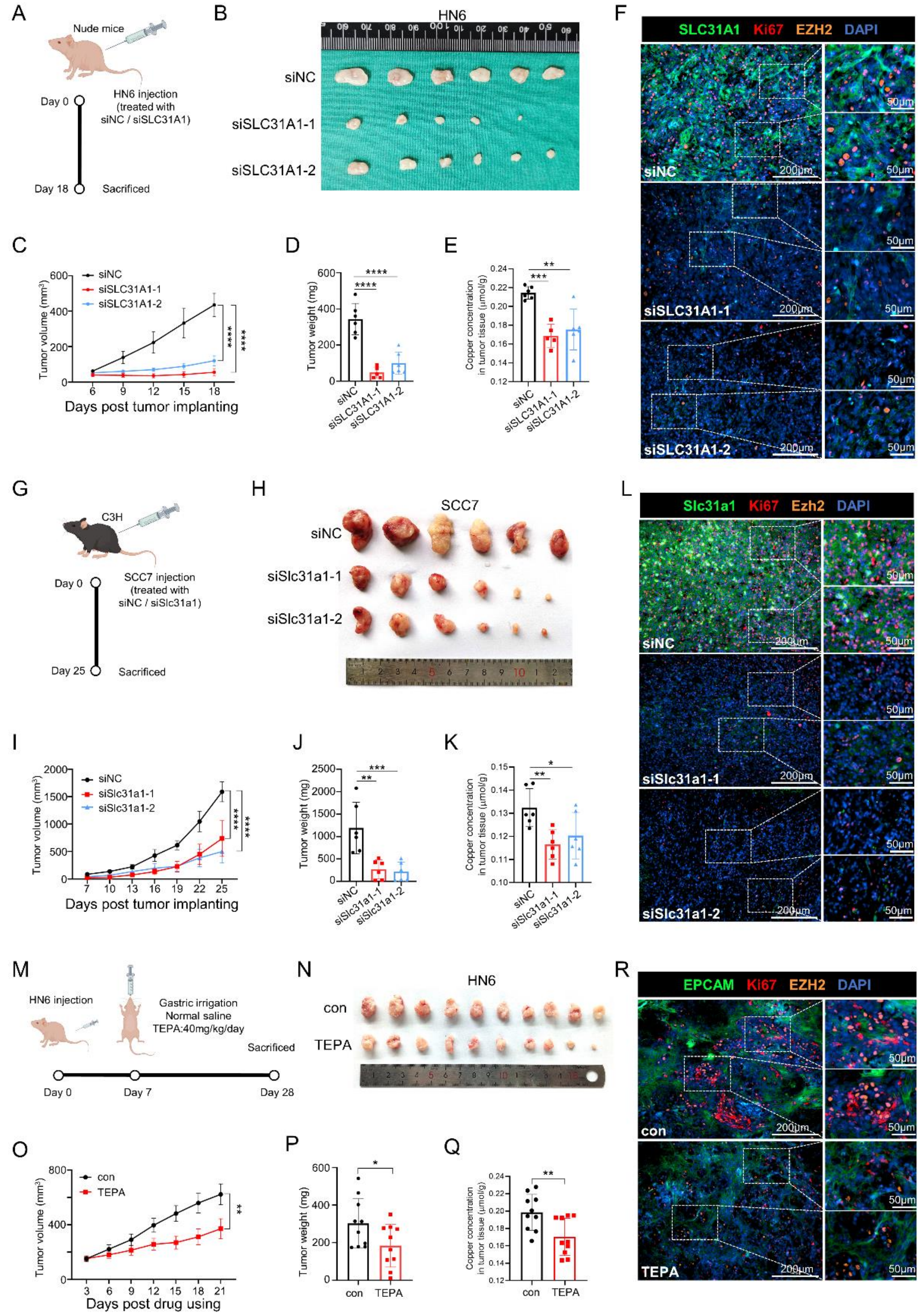

Figure S5. (**A**) Schematic diagram of subcutaneous tumor (HN6) experiment in nude mice. (**B**) Macroscopic view of subcutaneous xenografts in nude mice. (**C**-**E**) Volume changes (**C**), weight (**D**) and copper concentration (**E**) of subcutaneous xenografts in nude mice. (**F**) Multiplexed

immunofluorescence of SLC31A1, Ki67 and EZH2 in subcutaneous xenograft tissues from nude mice, Scale bars, 200 μm (left), 50μm (right). (**G**) Schematic diagram of subcutaneous tumor (SCC7) experiment in C3H mice. (**H**) Macroscopic view of subcutaneous xenografts in C3H mice. (**I**-**K**) Volume changes (**I**), weight (**J**) and copper concentration (**K**) of subcutaneous xenografts in C3H mice. (**L**) Multiplexed immunofluorescence of Slc31a1, Ki67 and Ezh2 in subcutaneous xenograft tissues from C3H mice, Scale bars, 200 μm (left), 50μm (right). (**M**) Schematic diagram of subcutaneous tumor (HN6) and oral administration experiment in nude mice. (**N**) Macroscopic view of subcutaneous xenografts in nude mice. (**O**-**Q**) Volume changes (**O**), weight (**P**) and copper concentration (**Q**) of subcutaneous xenografts in nude mice. (**R**) Multiplexed immunofluorescence of EPCAM, Ki67 and EZH2 in subcutaneous xenograft tissues from nude mice, Scale bars, 200 μm (left), 50μm (right). Data in C-E, I-K and O-Q were calculated by two-tailed unpaired Student's t test.

Figure S6

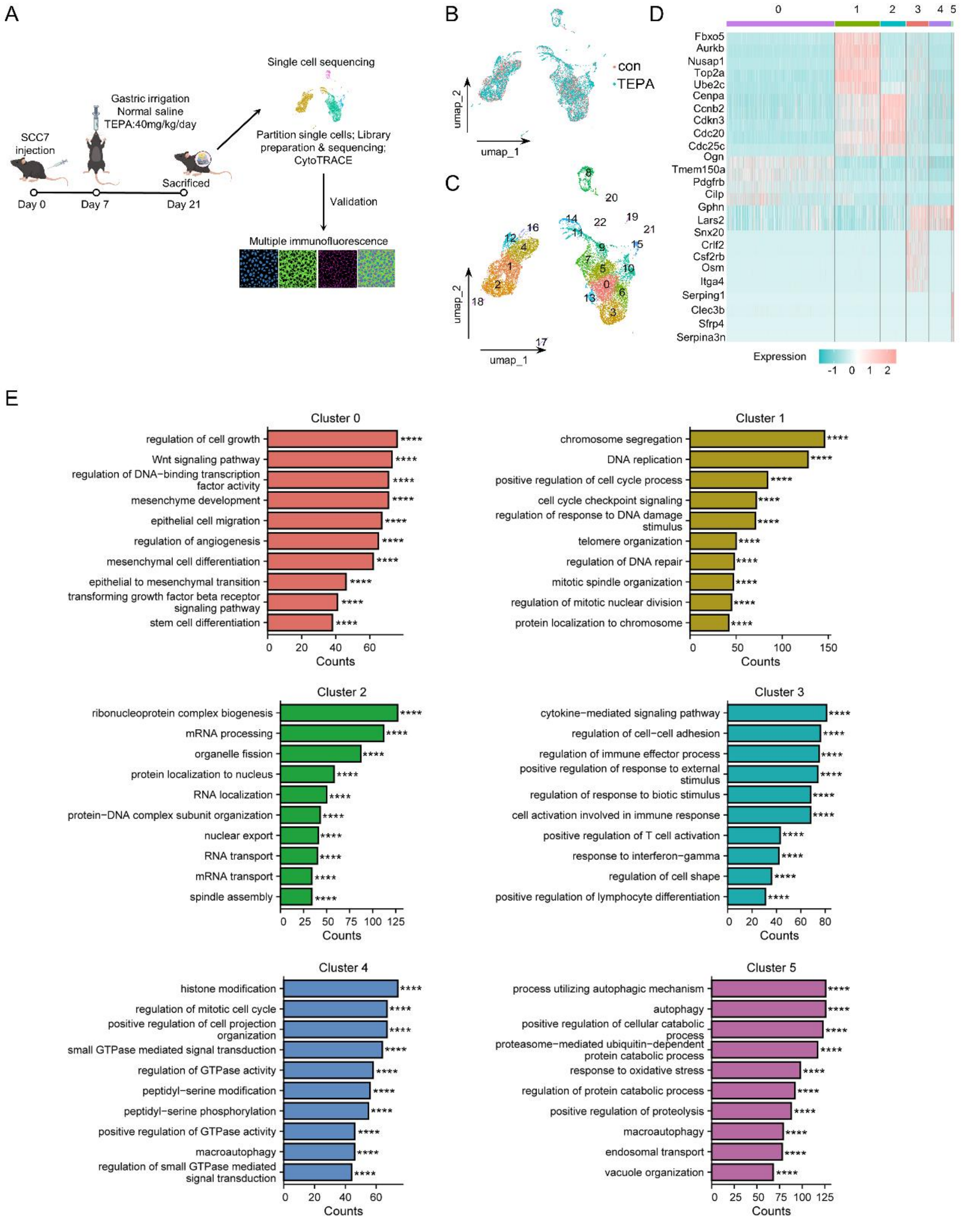

Figure S6. (**A**) Schematic diagram of the scRNA-seq analysis process for subcutaneous xenograft tumors (SCC7) in C3H mice. (**B-C**) Dimensionality reduction clustering analysis of cells in subcutaneous xenograft tumors in C3H mice. (**D**) Subgrouping of tumor epithelial cells in transplanted tumors of C3H mice based on marker genes. (**E**) Functional enrichment analysis of marker genes for various tumor epithelial cell clusters.

Figure S7

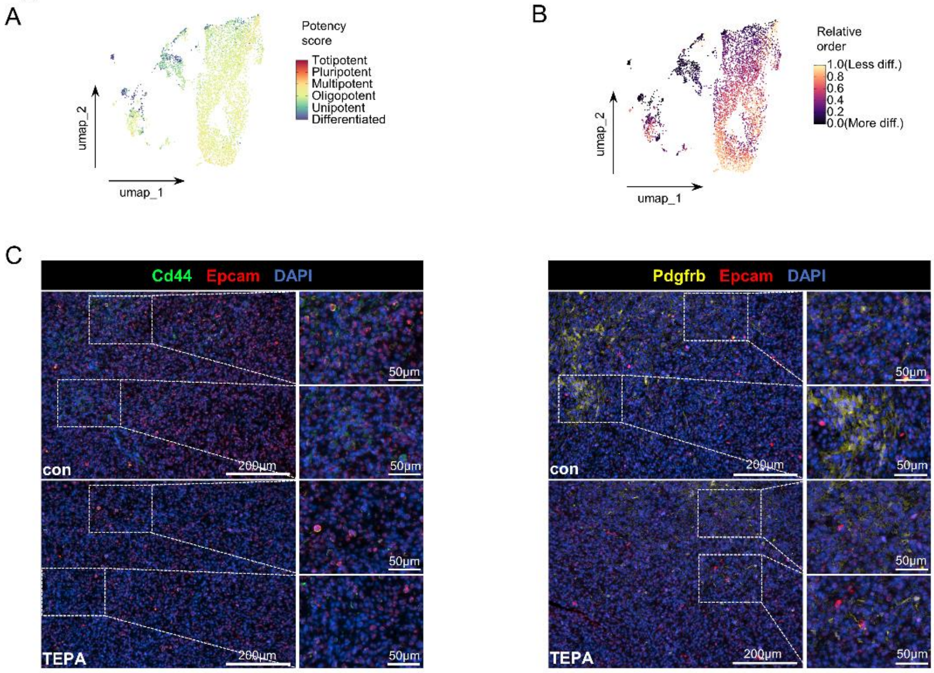

Figure S7. (**A**-**B**) CytoTRACE analysis of tumor epithelial cell. (**C**) Multiplexed immunofluorescence of Cd44, Pdgfrb and Epcam in subcutaneous xenograft tissues from C3H mice, Scale bars, 200 μm (left), 50μm (right).

Figure S8

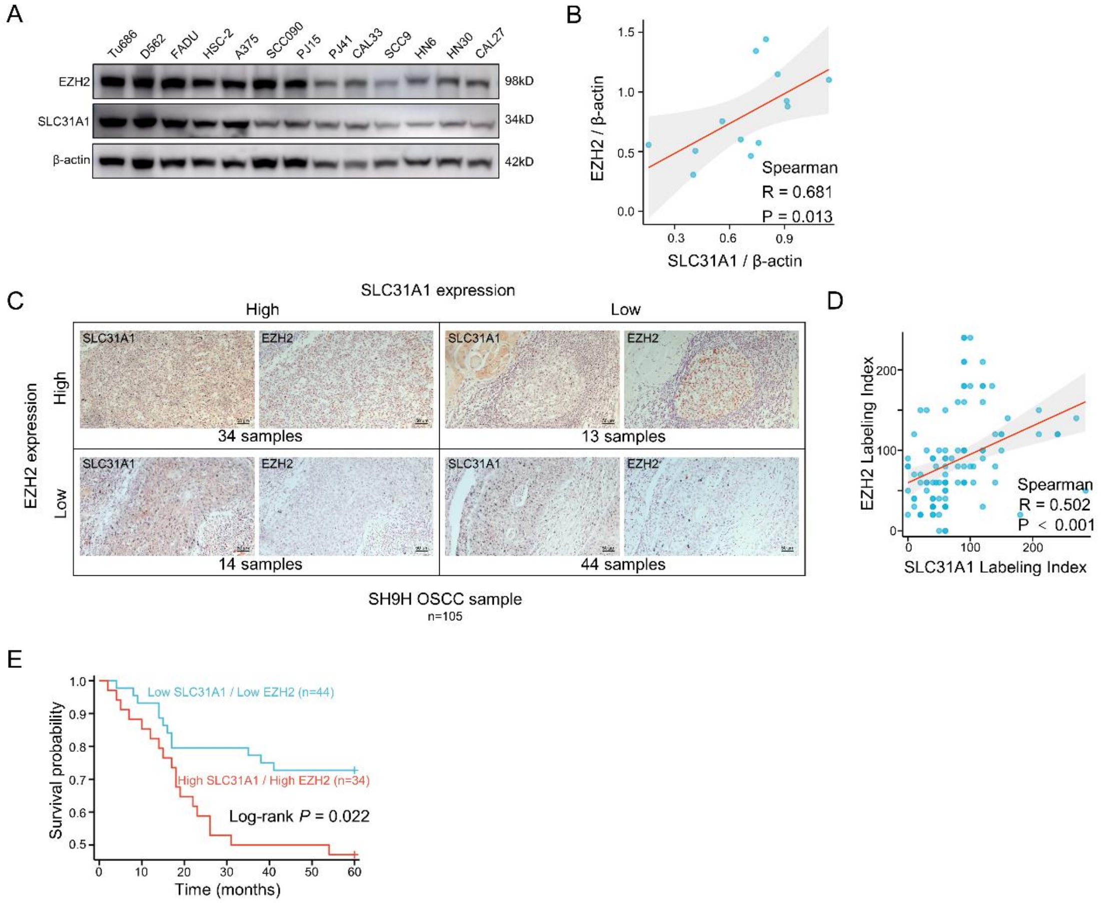

Figure S8. (**A**) Western blot experiments detected the protein expression levels of EZH2 and SLC31A1 in OSCC cell lines. (**B**) The correlation between EZH2 and SLC31A1 protein expression levels in OSCC cell lines was examined. (**C**) EZH2 and SLC31A1 expression were assessed in independent clinical samples of OSCC from Shanghai Ninth People's Hospital (n = 105), Scale bars, 50 μm. (**D**) The correlation between EZH2 and SLC31A1 protein expression levels in independent clinical samples of OSCC from Shanghai Ninth People's Hospital was analyzed. (**E**) The association between co-expression of EZH2 and SLC31A1 and patient prognosis was investigated in independent clinical samples of OSCC from Shanghai Ninth People's Hospital. Data in E was analyzed by Kaplan–Meier plots, p values were determined by a two-tailed log-rank test. Data in B and D were analyzed by Spearman Correlation Analysis.

Figure S9

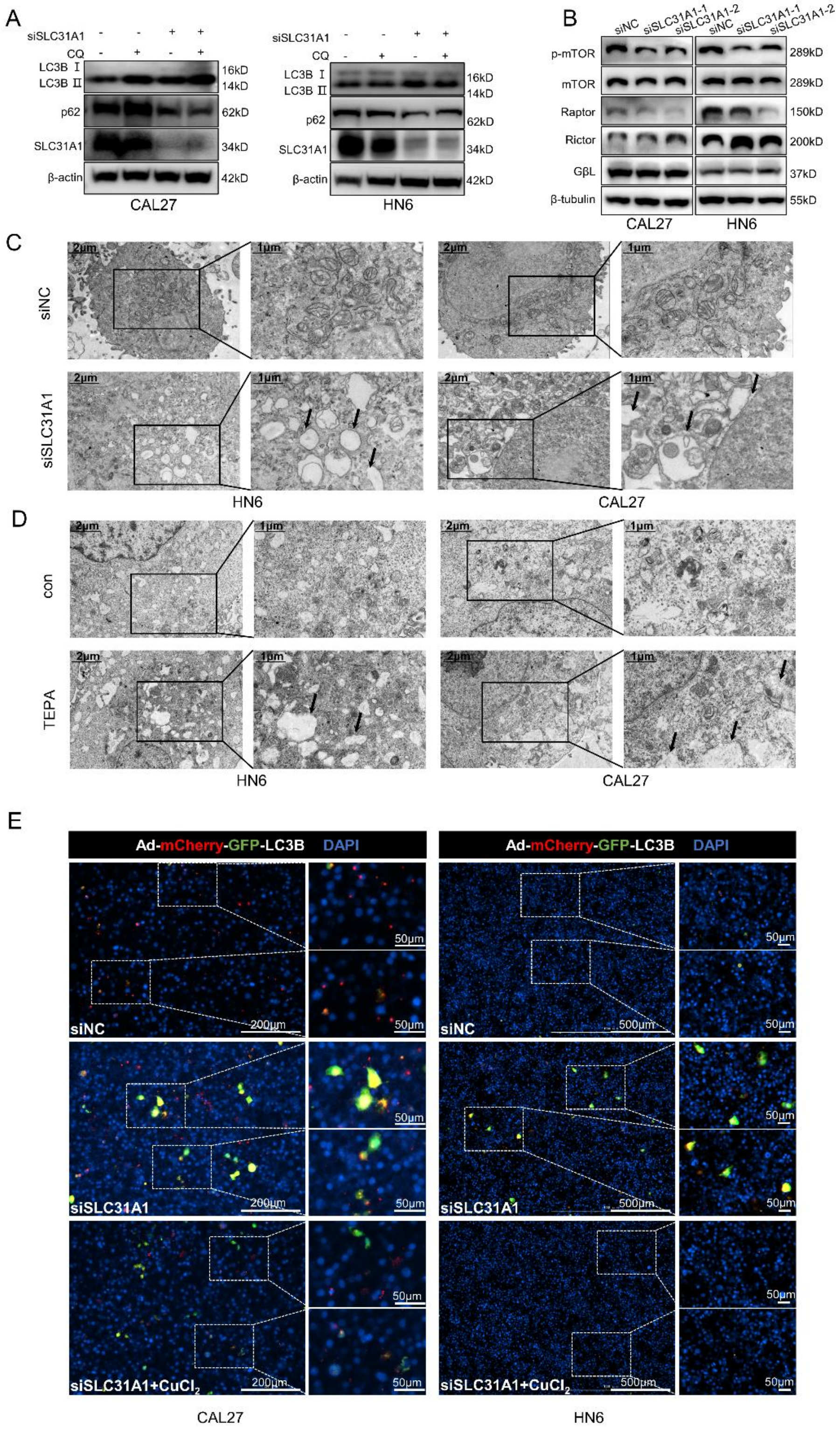

Figure S9. (**A**) Differential expression of LC3B and p62 proteins with or without CQ treatment detected by Western blot in CAL27 and HN6 cells after SLC31A1 silencing. (**B**) Differential expression of mTOR pathway proteins detected by Western blot in CAL27 and HN6 cells after

SLC31A1 silencing. (**C**) Detection of autophagosome formation after SLC31A1 silencing in HN6 and CAL27 cells, Scale bars, 2 μm (left), 1 μm (right). (**D**) Detection of autophagosome formation after treatment with copper chelator TEPA in HN6 and CAL27 cells, Scale bars, 2 μm (left), 1 μm (right). (**E**) Differential expression of LC3B with or without copper ion recovery detected after SLC31A1 silencing in CAL27 and HN6 cells, Scale bars, 200 μm (left), 50 μm (right).

Figure S10

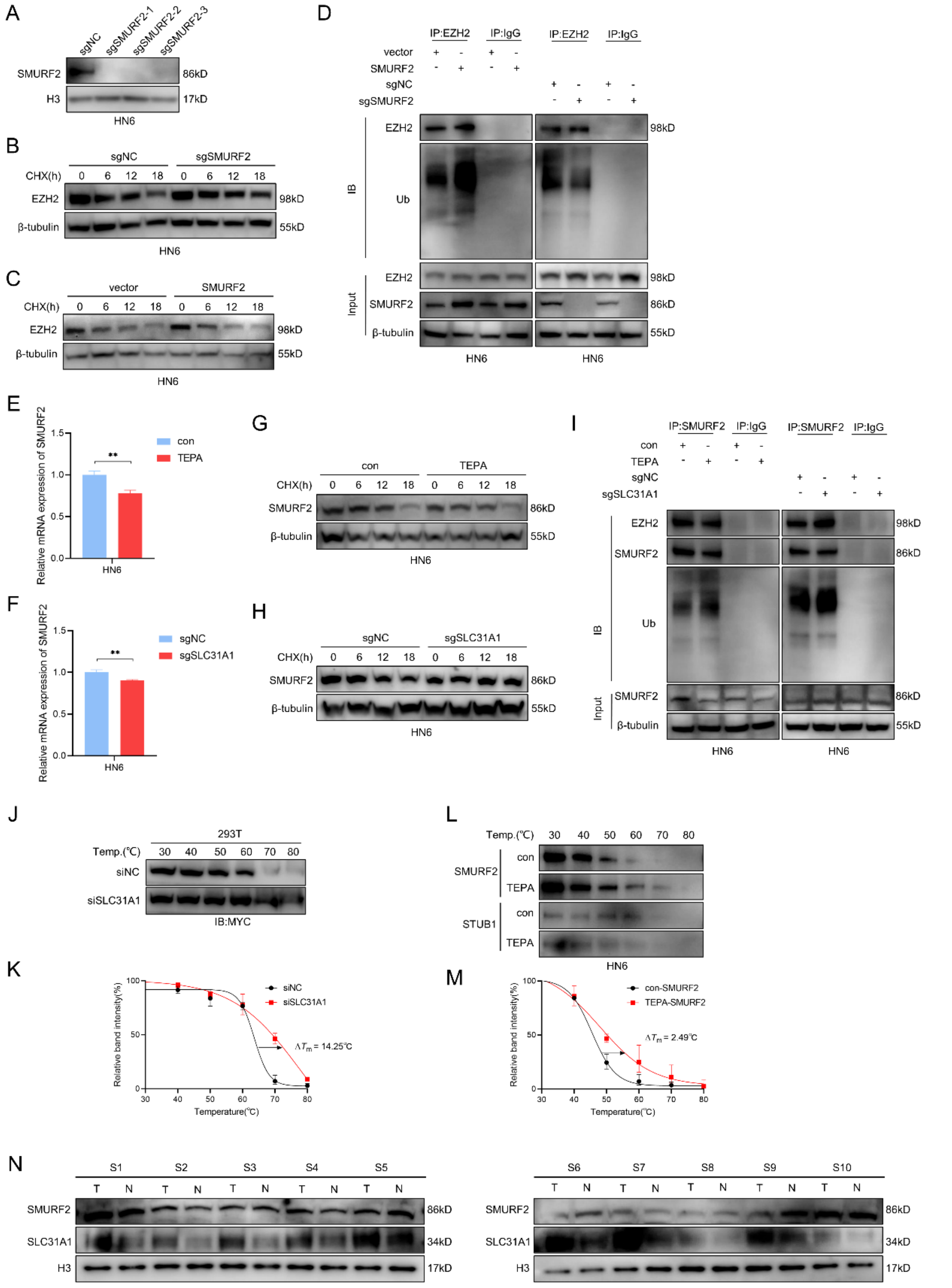

Figure S10. (**A**) Detection of SMURF2 protein expression levels in SMURF2-KO HN6 cells. (**B-C**) Detection of EZH2 protein expression levels in SMURF2-KO (**B**) and SMURF2-overexpression (**C**) HN6 cells treated with CHX (20 μg / mL) (0 h, 6 h, 12 h, 18 h). (**D**) Detection

of the EZH2 ubiquitination level in SMURF2-KO and SMURF2-overexpression HN6 cells. (**E**) Detection of the SMURF2 mRNA level in TEPA-treated HN6 cells. (**F**) Detection of the SMURF2 mRNA level in SLC31A1-KO HN6 cells. (**G**-**H**) Detection of SMURF2 protein expression levels in TEPA-treated (**G**) and SLC31A1-KO (**H**) HN6 cells treated with CHX (20 μg / mL) (0 h, 6 h, 12 h, 18 h). (**I**) Detection of the SMURF2 ubiquitination level in TEPA-treated and SLC31A1-KO HN6 cells. (**J-K**) Detection of SMURF2 thermal stability treated with SLC31A1-silenced via Cellular Thermal Shift Assay (CETSA) in 293T cell. (**L-M**) Detection of SMURF2 and STUB1 thermal stability via CETSA in TEPA-treated HN6 cell. (**N**) Expression levels of SMURF2 and SLC31A1 protein in 10 pairs of OSCC tumor tissues and adjacent normal tissues. Data in E-F were calculated by two-tailed unpaired Student's t test; Data in K and M were calculated by nonlinear regression (curve fit).

Figure S11

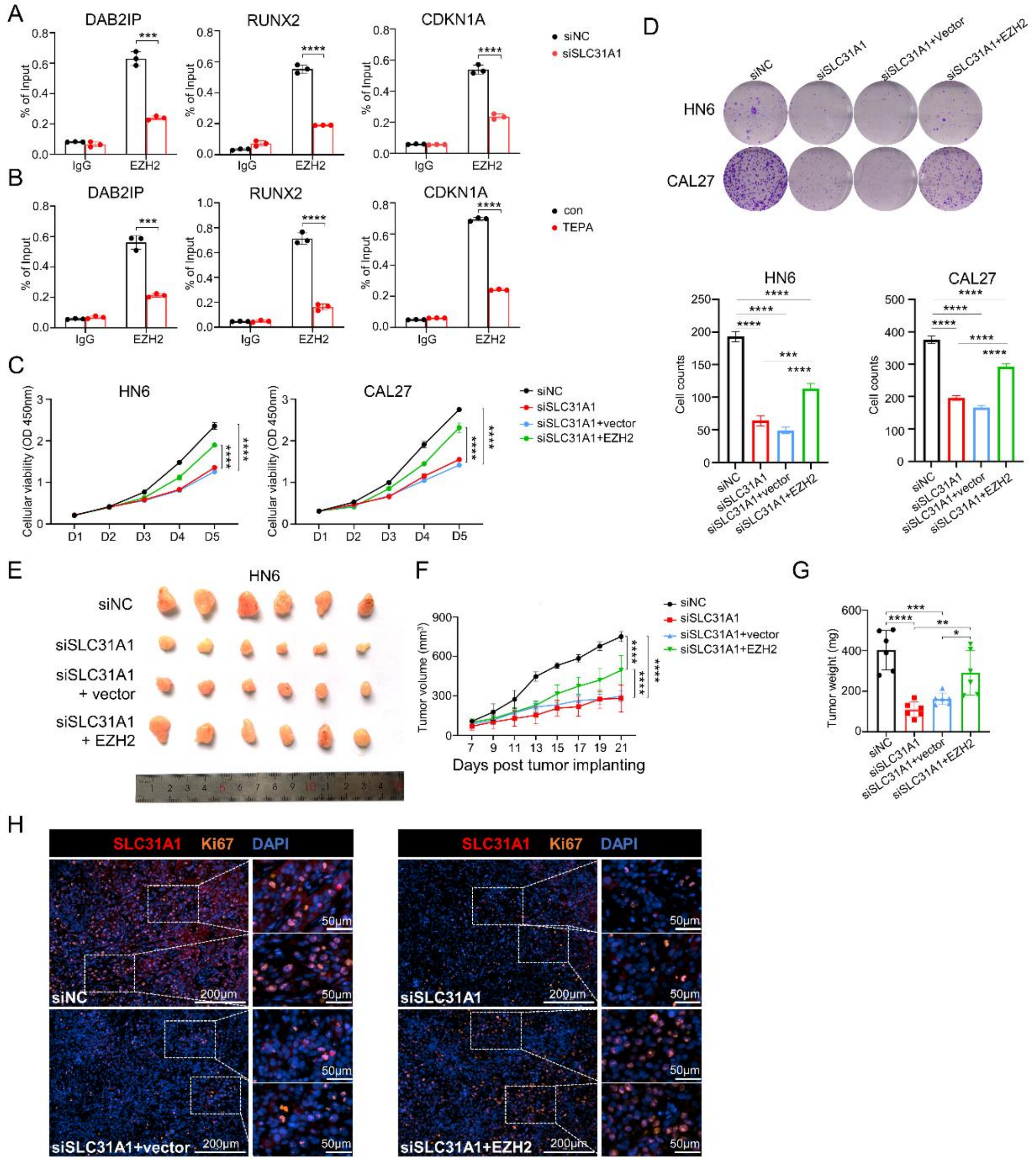

Figure S11. (**A**-**B**) ChIP-qPCR experiments detected the changes in enrichment of EZH2 protein on the promoter regions of EZH2 downstream target genes (DAB2IP, RUNX2 and CDKN1A) in SLC31A1-silenced (**A**) and TEPA-treated (**B**) CAL27 cells. (**C**) In OSCC cell lines (HN6 and CAL27) silenced for SLC31A1 and rescued EZH2 expression, changes in tumor cell proliferation were evaluated by CCK-8 assay. (**D**) In OSCC cell lines (HN6 and CAL27) silenced for SLC31A1 and rescued EZH2 expression, changes in tumor cell clonogenic ability were evaluated by colony formation assay. (**E**) Macroscopic view of subcutaneous xenografts (HN6)

in nude mice. (**F**) Changes in volume of subcutaneous xenografts in nude mice. (**G**) Weight of subcutaneous xenografts in nude mice. (**H**) Multiplexed immunofluorescence of SLC31A1 and Ki67 in subcutaneous xenograft tissues from nude mice, Scale bars, 200 μm (left), 50 μm (right). Data in A-D, F-G were calculated by two-tailed unpaired Student's t test.

Figure S12

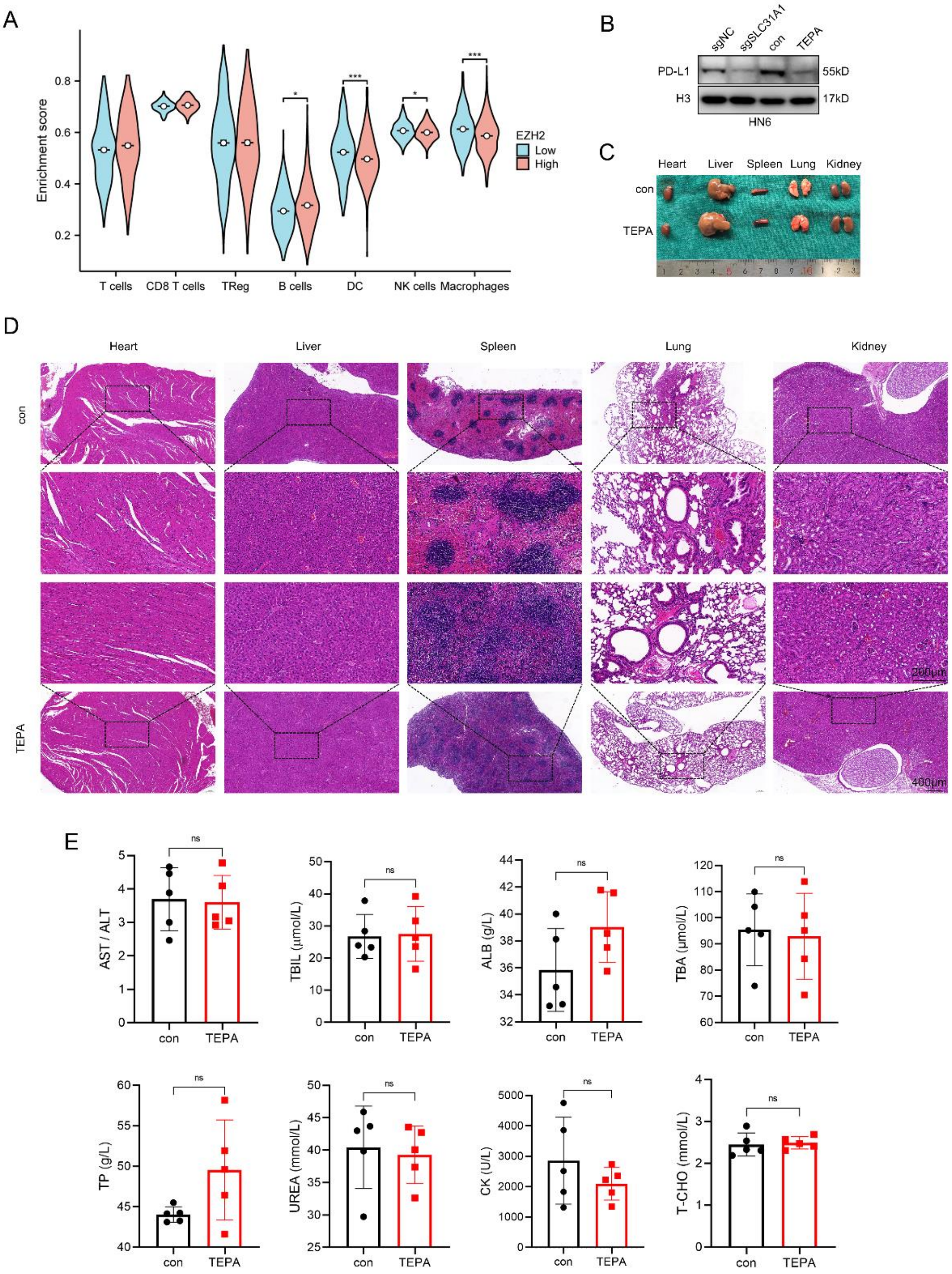

Figure S12. (**A**) Enrichment score of immune cells in EZH2 low and high groups in TCGA-OSCC. (**B**) Detection of PD-L1 protein expression levels in HN6 cells after copper starvation. (**C**) Macroscopic view of heart, liver, spleen, lung and kidney in C3H. (**D**) HE staining of heart, liver, spleen, lung and kidney in C3H, Scale bars, 400 μm, 200μm. (**E**) Detection of serum biochemical assays for liver and kidney function markers of C3H. Data in E were calculated by

two-tailed unpaired Student’s t test.

Figure S13

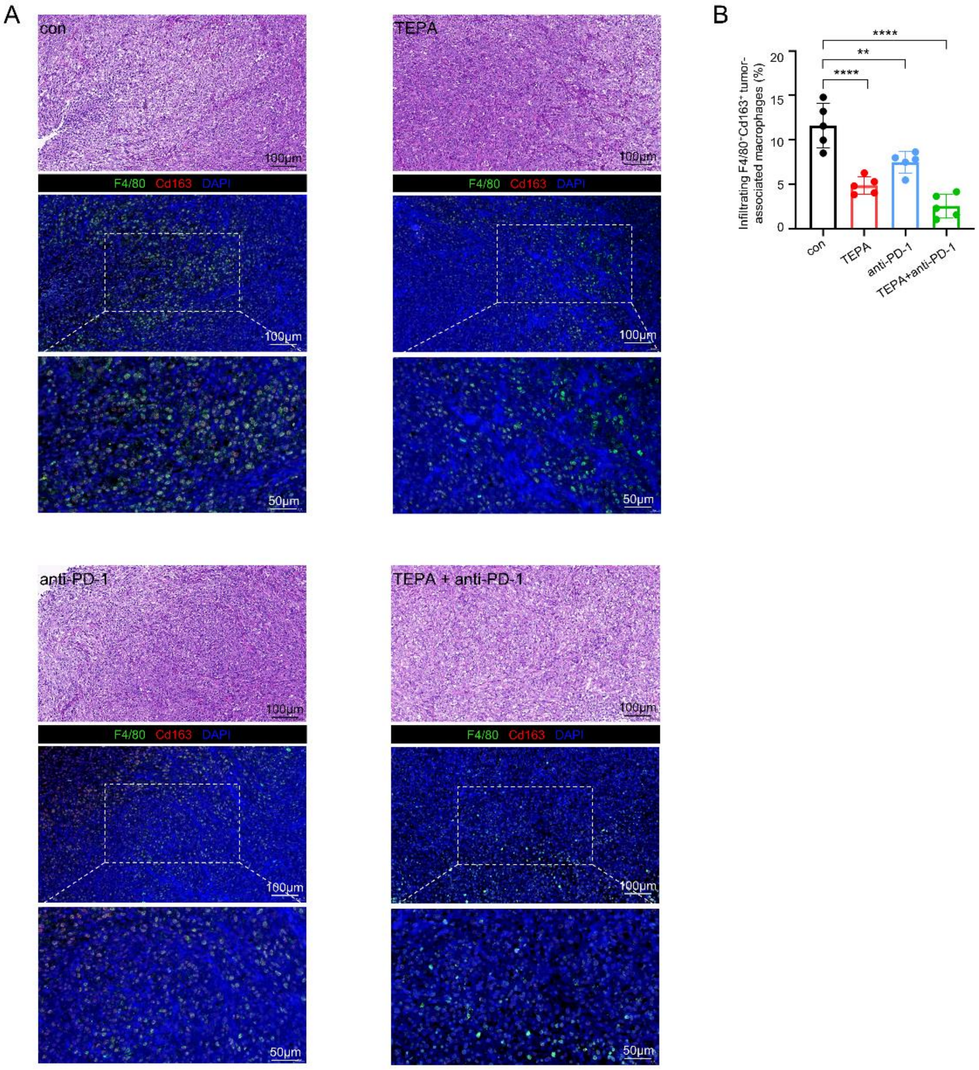

Figure S13. (**A**) HE staining and multiplexed immunofluorescence of F4/80 and Cd163 in subcutaneous xenograft tissues from C3H mice, Scale bars, 100 μm, 50μm. (**B**) The percentage of F4/80^{+}Cd163^{+} tumor-associated macrophages in C3H mice.

Table S1

Table S1 Primers used for qRT-PCR		
**ID**	**Primer**	**5' to 3'**
SLC31A1	F	GGGGATGAGCTATATGGACTCC
SLC31A1	R	TCACCAAACCGGAAAACAGTAG
Slc31a1	F	GAACCACACGGACGACAACAT
Slc31a1	R	CTCCACCGTGGGAGTGTGAG
EZH2	F	AATCAGAGTACATGCGACTGAGA
EZH2	R	GCTGTATCCTTCGCTGTTTCC
Ezh2	F	CGAATAACAGTAGCAGACCCAG
Ezh2	R	TGTTTGACACCGAGAATTTGCTT
18s	F	CGCCGCTAGAGGTGAAATTCT
18s	R	CGAACCTCCGACTTTCGTTCT
ACTB	F	CATGTACGTTGCTATCCAGGC
ACTB	R	CTCCTTAATGTCACGCACGAT
Actb	F	GTGACGTTGACATCCGTAAAGA
Actb	R	GCCGGACTCATCGTACTCC
TP53	F	GAGGTTGGCTCTGACTGTACC
TP53	R	TCCGTCCCAGTAGATTACCAC
NOTCH1	F	GAGGCGTGGCAGACTATGC
NOTCH1	R	CTTGTACTCCGTCAGCGTGA
CDKN2A	F	GATCCAGGTGGGTAGAAGGTC

CDKN2A	R	CCCCTGCAAACTTCGTCCT
PTEN	F	TTTGAAGACCATAACCCACCAC
PTEN	R	ATTACACCAGTTCGTCCCTTTC
PIK3CA	F	CCACGACCATCATCAGGTGAA
PIK3CA	R	CCTCACGGAGGCATTCTAAAGT
SMURF2	F	GGCAATGCCATTCTACAGATACT
SMURF2	R	CAACCGAGAAATCCAGCACCT

Table S2

Table S2 Primers used for ChIP-qPCR		
**ID**	**Primer**	**5' to 3'**
CDKN1A	F	TTTTGTCCTTGGGCTGCCTG
CDKN1A	R	GCAGATCACATACCCTGTTCAG
RUNX2	F	CGTAGTAGTACACAACGCCG
RUNX2	R	GTTTCGTGTCTGTCTTCCCC
DAB2IP	F	CCTGCTTTCTGTTTCCTTCTCCTG
DAB2IP	R	TTGAACCACCTCCTCCTCCCTCTC
